# Comprehensive virtual screening of 4.8 k flavonoids reveals novel insights into allosteric inhibition of SARS-CoV-2 M^PRO^

**DOI:** 10.1038/s41598-021-94951-6

**Published:** 2021-07-29

**Authors:** Gabriel Jiménez-Avalos, A. Paula Vargas-Ruiz, Nicolás E. Delgado-Pease, Gustavo E. Olivos-Ramirez, Patricia Sheen, Manolo Fernández-Díaz, Miguel Quiliano, Mirko Zimic, COVID-19 Working Group in Perú, Andres Agurto-Arteaga, Ricardo Antiparra, Manuel Ardiles-Reyes, Katherine Calderon, Yudith Cauna-Orocollo, Maria de Grecia Cauti-Mendoza, Naer Chipana-Flores, Ricardo Choque-Guevara, Xiomara Chunga-Girón, Manuel Criollo-Orozco, Lewis De La Cruz, Elmer Delgado-Ccancce, Christian Elugo-Guevara, Manolo Fernández-Sanchez, Luis Guevara-Sarmiento, Kristel Gutiérrez, Oscar Heredia-Almeyda, Edison Huaccachi-Gonzalez, Pedro Huerta-Roque, Eliana Icochea, Gisela Isasi-Rivas, Romina A. Juscamaita-Bartra, Abraham Licla-Inca, Angela Montalvan, Ricardo Montesinos-Millan, Dennis Núñez-Fernández, Adiana Ochoa-Ortiz, Erika Páucar-Montoro, Kathy Pauyac, Jose L. Perez-Martinez, Norma Perez-M, Astrid Poma-Acevedo, Stefany Quiñones-Garcia, Ingrid Ramirez-Ortiz, Daniel Ramos-Sono, Angela A. Rios-Angulo, Dora Rios-Matos, Aldo Rojas-Neyra, Yomara K. Romero, Mario I. Salguedo-Bohorquez, Yacory Sernaque-Aguilar, Luis F. Soto, Luis Tataje-Lavanda, Julio Ticona, Katherine Vallejos-Sánchez, Doris Villanueva-Pérez, Freddy Ygnacio-Aguirre

**Affiliations:** 1 Laboratorio de Bioinformática, Biología Molecular y Desarrollos Tecnológicos, Facultad de Ciencias y Filosofía, Departamento de Ciencias Celulares y Moleculares, Universidad Peruana Cayetano Heredia (UPCH), 15102 Lima, Peru; 2 Farmacológicos Veterinarios - FARVET S.A.C. Chincha, Lima, Peru; 3 Faculty of Health Sciences, Centre for Research and Innovation, Universidad Peruana de Ciencias Aplicadas (UPC), 15023 Lima, Peru

**Keywords:** Computational biology and bioinformatics, Drug discovery, Structural biology

## Abstract

SARS-CoV-2 main protease is a common target for inhibition assays due to its high conservation among coronaviruses. Since flavonoids show antiviral activity, several in silico works have proposed them as potential SARS-CoV-2 main protease inhibitors. Nonetheless, there is reason to doubt certain results given the lack of consideration for flavonoid promiscuity or main protease plasticity, usage of short library sizes, absence of control molecules and/or the limitation of the methodology to a single target site. Here, we report a virtual screening study where dorsilurin E, euchrenone a11, sanggenol O and CHEMBL2171598 are proposed to inhibit main protease through different pathways. Remarkably, novel structural mechanisms were observed after sanggenol O and CHEMBL2171598 bound to experimentally proven allosteric sites. The former drastically affected the active site, while the latter triggered a hinge movement which has been previously reported for an inactive SARS-CoV main protease mutant. The use of a curated database of 4.8 k flavonoids, combining two well-known docking software (AutoDock Vina and AutoDock4.2), molecular dynamics and MMPBSA, guaranteed an adequate analysis and robust interpretation. These criteria can be considered for future screening campaigns against SARS-CoV-2 main protease.

## Introduction

The coronavirus disease-2019 (COVID-19), caused by the severe acute respiratory syndrome coronavirus 2 (SARS-CoV-2), was first reported in late December 2019 in Wuhan, People’s Republic of China. As of June 18th, 2021, there have been over 177.7 million cases of COVID-19 and over 3.8 million deaths reported globally^1^. Developing effective treatments is a priority among the scientific community. This objective requires systematic narrowing of drug candidates, usually by virtual screening methods^2,3^.

One of the most widely studied SARS-CoV-2 proteins is the main protease (M^PRO^). M^PRO^ is responsible for the catalytic cleavage of peptides necessary for viral replication and transcription^4,5^. In addition, the human proteome has no protein homologous to M^PRO^, which is advantageous for the search of an effective inhibitor^6^. Therefore, SARS-CoV-2 M^PRO^ is a suggested target for virtual screening efforts^7,8^.

Most screenings efforts against M^PRO^ have focused on its active site. However, this approach could prove too restrictive since allosteric inhibition and dimerization disruption are plausible alternatives^9–13^. Recent in vitro research has shown that many substances could inhibit M^PRO^ by binding regions far from the active site^9,10^. Hence, identifying allosteric sites and targeting them with ample libraries may increase target proteins’ druggability^14,15^. Nonetheless, the dynamics of the allosterisms observed in vitro are not completely clear. For this reason, in silico studies could shed light on the mechanisms behind them.

Despite its importance, no in silico research has directed a massive drug screening towards the discovery of allosteric inhibitors of M^PRO^. An explanation for this absence could be the difficulty in recognizing allosteric sites. These regions are usually cryptic, as they cannot be easily visualized in an *apo*-structure^14^. Fortunately, the recent development of tools such as CryptoSite^14^ greatly facilitates the detection of these so-called “cryptic sites”. Given the existence of these tools and the evidence of allosteric inhibition in M^PRO^, there is good reason to expand research beyond the active site.

Flavonoids are a group of molecules with in vitro inhibitory activity against SARS-CoV-2 M^PRO^^16^. As naturally occurring substances, flavonoids are an important source of treatment in biodiverse countries. Besides, natural products may be more easily accepted as medicine than artificial ones^17^. Furthermore, flavonoids are polycyclic and hydrophobic. Both properties are recurring characteristics among active site inhibitors^18,19^. Hence, the rich online flavonoids databases could hold promising candidates for COVID-19 treatment.

Because of their potential, several in silico studies have screened flavonoids against M^PRO^^20–26^. The reliability of these studies is put into question for three reasons. (1) They lack pan-assay interference compounds (PAINS) filtering. Some flavonoids tend to behave as PAINS^27^ and should be purged to prevent false positives^28–30^. From the conventional flavonoid docking/molecular dynamics screening works^20–25^, only one was filtered for possible PAINS^25^. (2) They lack proper positive controls. Three published pieces used positive controls^21,22,24^. One did not report results related to theirs. The remaining two employed the redocking method^21,22^. Nonetheless, redocking overestimates the experiment’s quality, which is why cross-docking is recommended instead^31^. Moreover, one study using glycosylated flavonoids concluded its positive control was successful^22^. However, it presented a picture where the docked ligand differs markedly from the real binding mode. (3) They lack negative controls. A screening protocol must show it can reject inert molecules, especially when working with libraries that contain ligands with possible false positives. Additionally, the studies that worked exclusively with flavonoids used databases of less than 200 compounds^21,22,24,25^.

The present study seeks to avert previous shortcomings by generating an extensive, filtered flavonoid database. The database will be used for a virtual screening directed at more than one SARS-CoV-2 M^PRO^ site. Virtual screening results will be coupled with unbiased molecular dynamics and proper rescoring by Molecular Mechanics Poisson-Boltzmann Surface Area (MMPBSA) to filter false positives^32^. The present work will be the first to attempt a massive flavonoid screening for allosteric sites. Thus, flavonoids reported by the present article and the structural mechanisms related to them could be a starting point for designing new molecules to combat SARS-CoV-2.

## Results

### M^PRO^ putative binding sites

**Figure 1:**
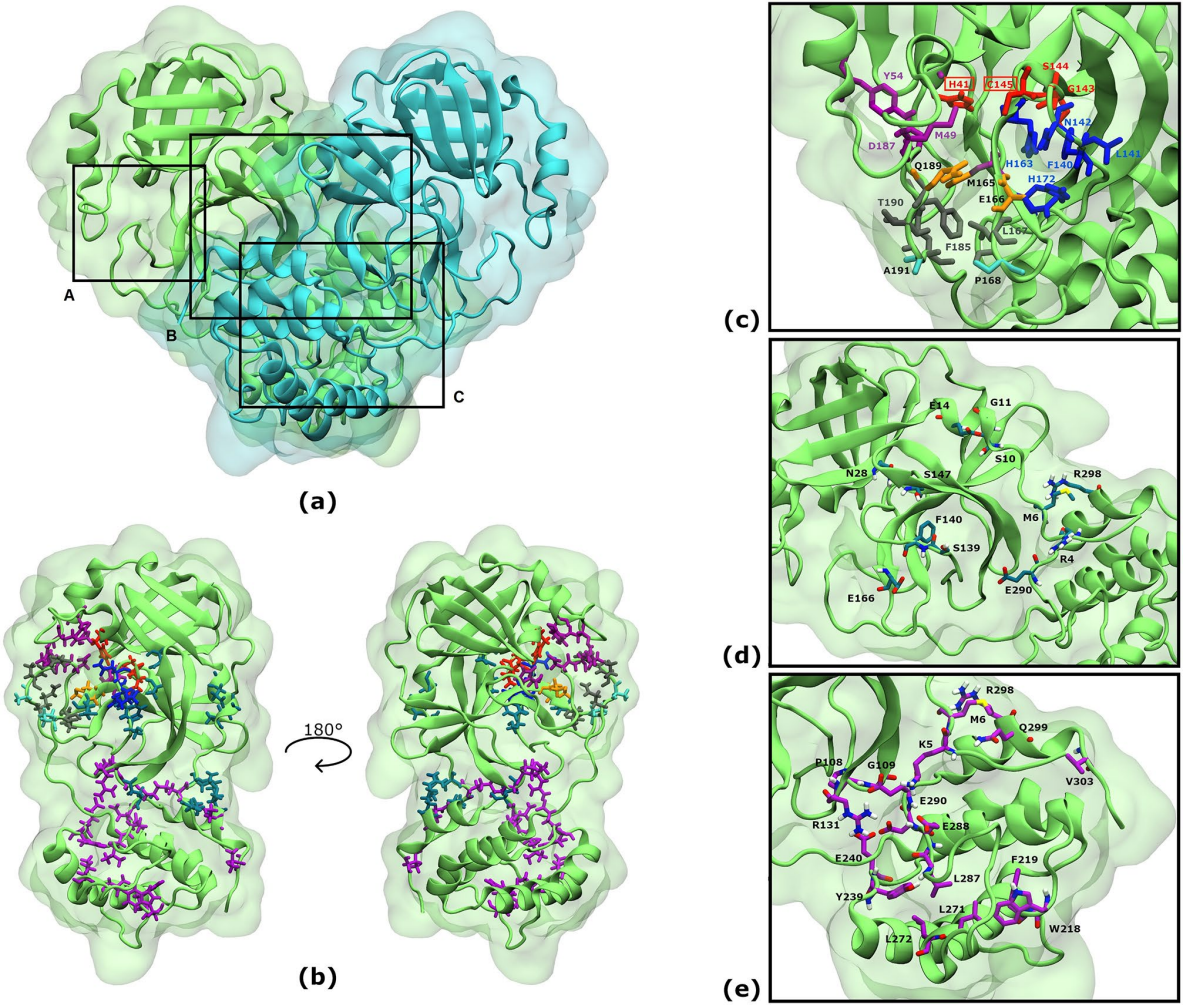
SARS-CoV-2 M^PRO^ and its three Putative Binding Sites (PBS). (**a**) Protomers are in cartoon and surface representations, each in a different colour; black boxes indicate the position of PBSs: (A) Substrate Binding Site, (B) Dimer Site, (C) Cryptic Site. (**b**) Residues conforming the binding sites are depicted in the green protomer as sticks according to colour code: blue for dimerization and magenta for cryptic binding sites, respectively. Substrate binding site residues and labels are coloured according to 5 different subregions: red (S1’: His41, Gly143, Ser144 and Cys145^13,35^), blue (S1: Ser1 (from the other protomer, not shown), Phe140, Leu141, Asn142, His163, Glu166 and His172^34^), purple (S2: His41, Met49, Tyr54, Met165 and Asp187^34^), orange (S3: Met165, Glu166 and Gln189^11^), grey (S4: Leu167, Pro168, Phe185, Thr190, Ala191, Gln192 and Gln189^34,36^) and cyan (S5: Pro168, Ala191^34^). Residues with two colours belong to two subregions; in this case, text labels are coloured black. (**c**) Close-up view of SBS. Labels from catalytic residues are enclosed in a red rectangle. (**d**) Close-up view of DS. DS residues were defined according to the literature^7^: Arg4, Met6, Ser10, Gly11, Glu14, Asn28, Ser139, Phe140, Ser147, Glu166, Glu290 and Arg298. (**e**) Close-up view of CS. CS residues were predicted using CryptoSite: Lys5, Met6, Pro108, Gly109, Arg131, Trp218, Phe219, Tyr239, Glu240, Leu271, Leu272, Leu287, Glu288, Asp289, Glu290, Arg298, Gln299 and Val303.

M^PRO^ is a homodimer (Fig. 1a). It consists of three domains: I (residues 10–99), II (residues 100–182) and III (residues 198–303)^13^. Domains II and III are joined by a flexible connecting loop (residues 183–197).

Targeting substrate binding sites (SBSs) is a common strategy for virtual screenings (VSs)^7,13,33^. Hence, flavonoids were first docked against this region. SBS residues were defined after bibliographic consideration (Fig. 1b,c)^11,13,34–36^.

M^PRO^ is inactive as a monomer^9,10,12,13^. Therefore, VS also targeted the dimerization site (DS) as disrupting dimerization would impair M^PRO^. DS residues were defined according to the literature (Fig. 1b,d)^7^.

The present study also screened for flavonoids that could bind allosteric pockets, as recent evidence suggests M^PRO^ can be allosterically inhibited^9,10^. However, allosteric pockets are usually cryptic. CryptoSite^14^ was used to detect residues with high probability of being part of a “cryptic site” (CS) (Fig. 1b,e). SBS, DS and CS are collectively called putative binding sites (PBSs).

### Flavonoid structural database

The complete database was built through data mining and included 6697 compounds. 111 compounds were excluded because they had more than 32 torsions. Possible PAINS are listed in Supplementary Dataset 1 and were removed. The final database comprised 4858 compounds (Supplementary Dataset 2).

### Fast-ranking screening

As suggested by other studies^33,37–41^, VS was performed through two stages: a fast-ranking, followed by an exhaustive-ranking screening. The fast-ranking VS was performed in Autodock Vina v. 1.1.2 (Vina)^42^. Considering SBS plasticity^12,43,44^, the screening was conducted against two different conformations: a ligand-induced SBS (ISBS) and a ligand-free or normal SBS (NSBS). The dimeric biological assembly of each structure was employed for ISBS and NSBS targeting. For DS and CS, the unligated M^PRO^ (PDB ID: 6YB7)^45^ in its monomeric form was the chosen receptor.

Fast-ranking results are given in Supplementary Dataset 3a–d. From this list, the top 100 compounds for each PBS had mean binding energies of − 8.89, − 10.05, − 8.07 and − 9.12 kcal/mol for the NSBS, the ISBS, the DS and the CS, respectively. Only these top compounds were selected for the exhaustive-ranking stage.

### Exhaustive-ranking screening

The top 100 compounds for NSBS, ISBS, DS and CS were subjected to a second, exhaustive ranking. The exhaustive stage was conducted in the GPU-implemented version of AutoDock4.2 (AutoDock-GPU)^46^. Binding mode selection for each docking was performed by ranking them by 2Dscore^47^. This type of ranking allows selection of the conformation with the lowest energy and most populated cluster.

Exhaustive-ranking results by PBS are given as Supplementary Dataset 4a–d. After cross-referencing the NSBS and ISBS top 30, five compounds per PBS were retrieved (Supplementary Table 1). Binding modes, energies and interactions of the top 5 compounds are shown in Supplementary Fig. 1 and Supplementary Table 2.

All SBS-binding ligands interacted with one catalytic residue (His41, Supplementary Table 2). Dorsilurin E (FL3FQUNP0001) presented the best binding energy to both NSBS (− 9.51 kcal/mol) and ISBS (− 11.31 kcal/mol). It had mostly hydrophobic interactions and only one hydrogen bond. For DS, sanggenol O (FL2FALNP0020) was the best inhibitor with a − 7.29 kcal/mol binding energy. Lastly, CS ligand CHEMBL2171598 was the best inhibitor with a binding energy of − 10.59 kcal/mol.

All DS docked ligands inserted at least one aromatic ring into the same small pocket reported in previous studies to bind allosteric inhibitors^9–11^. The cavity will be referred to as region A (Supplementary Fig. 2a,b). CS ligands kurzichalcolactone (FL1CA9NC0001) and CHEMBL2171598 were found to bind region A in the same fashion as the DS docked ligands.

Out of the 15 compounds, 7 were ranked within the top 30 in both fast and exhaustive screenings (Supplementary Table 3). These included DS and CS best inhibitors. Notably, sanggenol O was ranked first in both stages.

### Positive and negative controls

**Figure 2:**
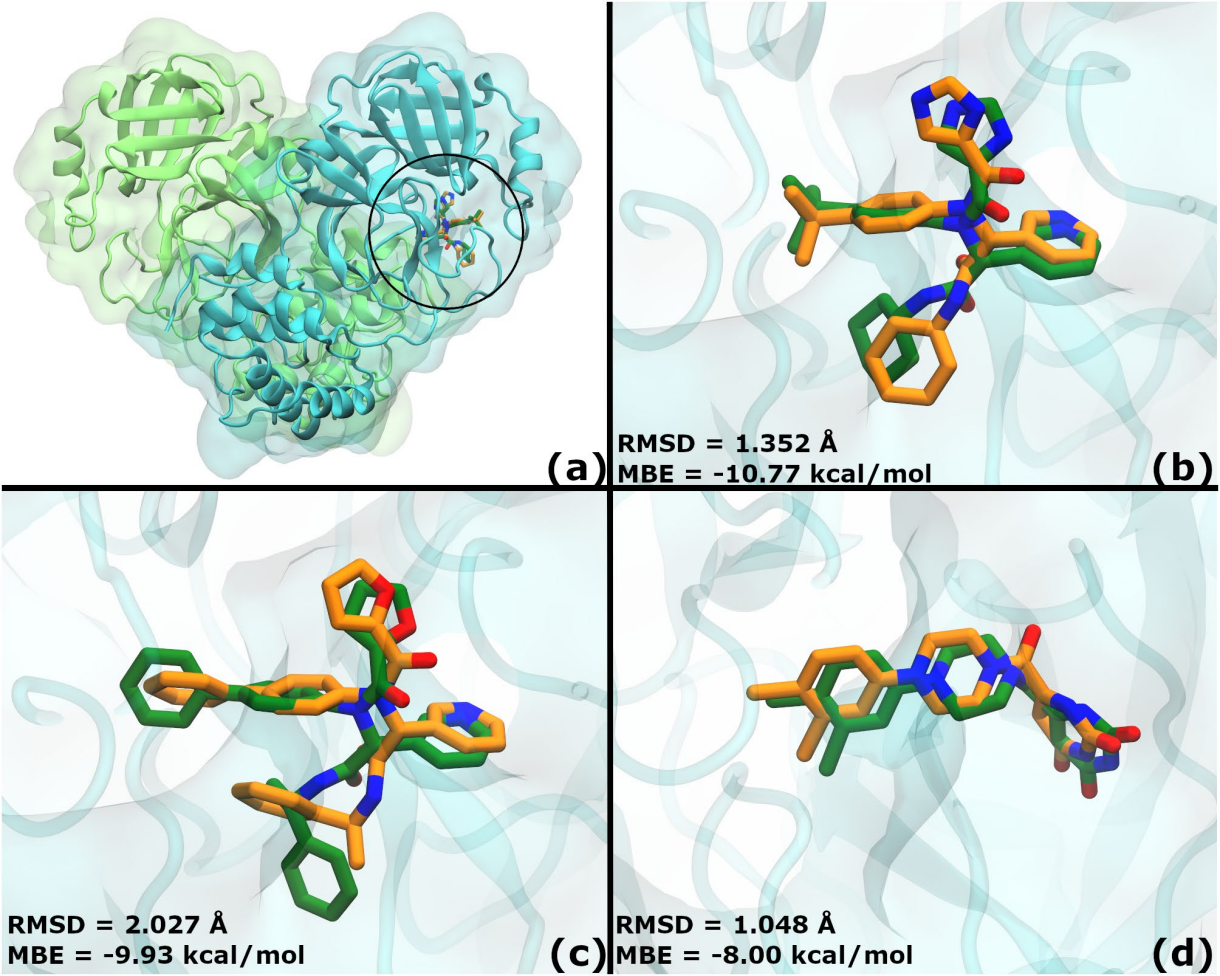
Structural alignment between positive controls and the original M^PRO^ crystal. Docked non-covalent inhibitors X77 (PDB ID: 6W63)^48^, X7V (PDB ID: 7KX5)^49^ and YD1 (PDB ID: 7LTJ)^50^ were aligned to their original M^PRO^ crystals. Docking was carried out following the exhaustive-ranking procedure using AutoDock-GPU. RMSD was calculated without considering hydrogen atoms. (**a**) Representative ligand X77 in the substrate binding site (black circle). All controls were also bound to this region. The M^PRO^ crystal is shown as in Fig. 1. Ligands are shown in sticks: orange for the control and green for the cross-docked inhibitors. Non-carbon atoms are coloured following the CPK colouring convention. Close-ups are provided to observe the docked pose in detail compared to the original position: (**b**) X77, RMSD = 1.352 Å, MBE = − 10.77 kcal/mol; (**c**) X7V, RMSD = 2.027 Å, MBE = -9.93 kcal/mol; (**d**) YD1, RMSD = 1.048 Å, MBE = − 8.00 kcal/mol. MBE, Mean Binding Energy. X77 was selected as the positive control for further analysis because it had the best docking energy.

A cross docking methodology was used for positive controls to validate the exhaustive-ranking stage. In that sense, non-covalent inhibitors X77, X7V and YD1 were excised from their corresponding complexes with M^PRO^ (PDB IDs: 6W63^48^, 7KX5^49^ and 7LTJ^50^, respectively) and docked against ISBS (Fig. 2a). In all cases, 2Dscore was used to select the binding mode. Structural comparison between these binding modes and the corresponding real conformations revealed high similarity for a cross docking assay (Fig. 2b–d). Root-mean-square deviation or RMSD (without hydrogens) were 1.352, 2.027 and 1.048 Å; while mean binding energies were − 10.77, − 9.93 and − 8.00 kcal/mol for X77, X7V and YD1, respectively.

Negative control TDZD-8 was docked against ISBS. The best pose according to the 2Dscore had an energy of − 5.46 kcal/mol. Considering the binding energies reported for the top 5 results for both NSBS and ISBS (Supplementary Dataset 4) and the binding energies of selected compounds against SBS (Supplementary Fig. 1), it was concluded that negative control was filtered by the exhaustive-ranking protocol.

### Molecular dynamics of *apo* protein structures

Molecular Dynamics (MD) of *apo* M^PRO^ (PDB ID: 6LU7)^34^ as a monomer and as a dimer were conducted for 600 ns. RMSD plots (Supplementary Fig. 3a) showed that dimeric M^PRO^ stabilizes at 120–130 ns, whereas monomeric M^PRO^ did so at nearly 290–310 ns. SBS residues only reached convergence in the dimer conformation, at around 2.9–3.1 Å (Supplementary Fig. 3b-c). Also, they were restrained in dimeric M^PRO^ (Supplementary Fig. 3d).

### Molecular dynamics of *holo* (protein–ligand) structures

MD of preliminary ligands with their corresponding receptors were computed for 600 ns. The docked complex between M^PRO^ and X77 was also subjected to 600 ns of simulations. This control was selected from the three because it had the best docking energy. For SBS, the complexes used were the ones from docking against ISBS, as they gave the best energies. Ligands that unbound during the first 100 ns were discarded.

**Figure 3:**
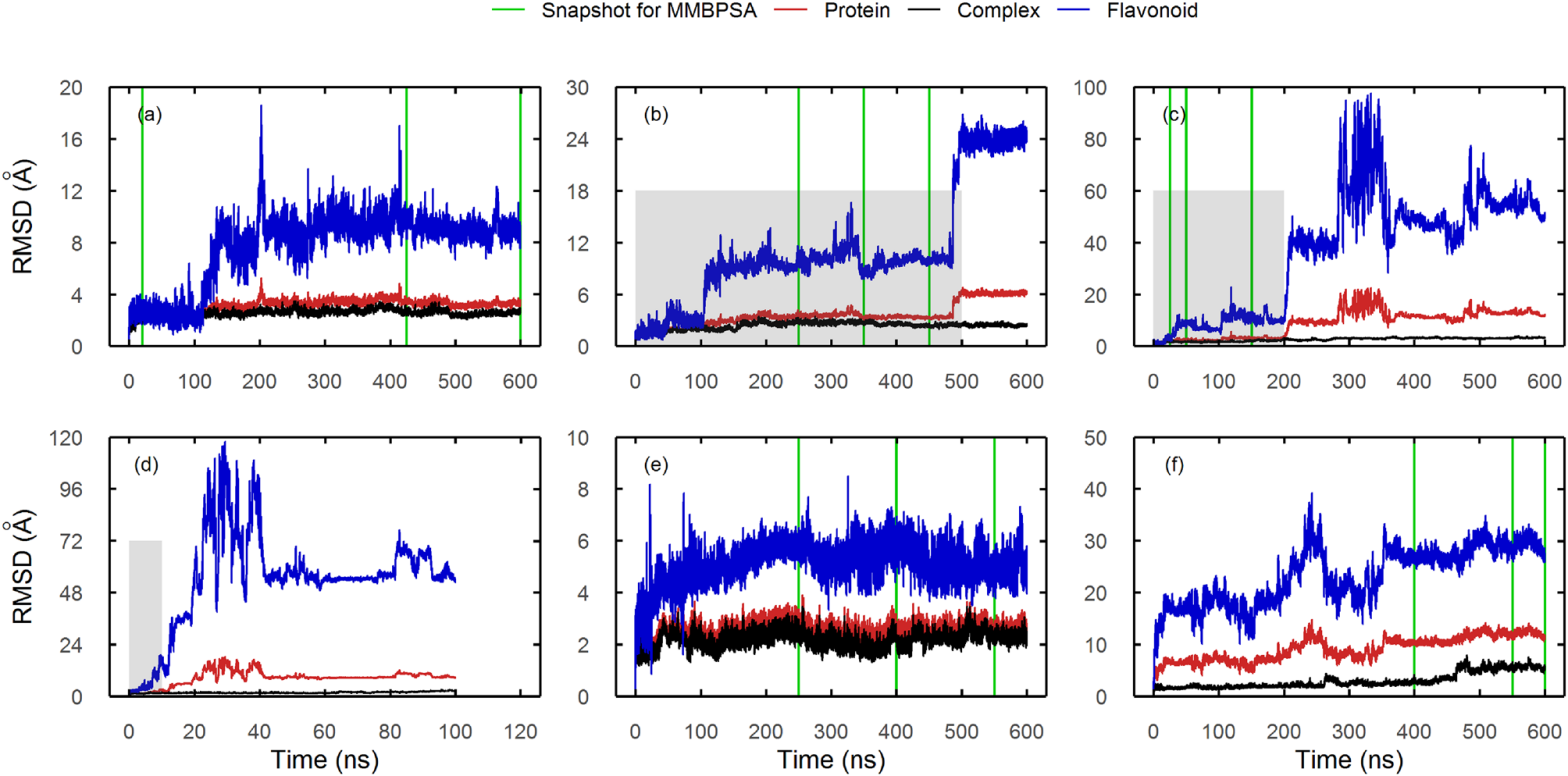
RMSD plots of flavonoid-M^PRO^ selected ligands along molecular dynamics simulations. Positive and negative controls are also shown. As negative control only remained associated during the first 10 ns, MD was not extended. RMSD of the protein, complex and flavonoid ligand is shown in red, black and blue, respectively. Green vertical lines reflect snapshots taken for further analysis with MMPBSA methods. The grey box indicates residence time (RT, time in which the ligand is interacting with the protein). In plots without a grey box, the ligand never detaches from the protein. *SBS*: (**a**) Dorsilurin E, (**b**) euchrenone a11 (detaches at 500 ns), (**c**) X77 (positive control, detaches at 200 ns), (**d**) TDZD-8 (negative control, detaches at 10 ns). In the SBS, the second lowest RMSD and fluctuations within RT pertained to dorsilurin E (< 5 Å during the first 100 ns, < 16 Å during all the RT, with exception of some spikes), which suggest stable binding. *DS*: (**e**) sanggenol O. *CS*: (**f**) CHEMBL2171598.

Regarding SBS compounds, dorsilurin E (Fig. 3a) had a bound residence time (RT) of 100%. It was followed by euchrenone a11 (FL2FALNP0014) (Fig. 3b) and X77 (83.3% and 33.3%, respectively) (Fig. 3c). Unlike previous ligands, TDZD-8 (negative control) (Fig. 3d) association only lasted for the first 10% of the MD.

**Figure 4:**
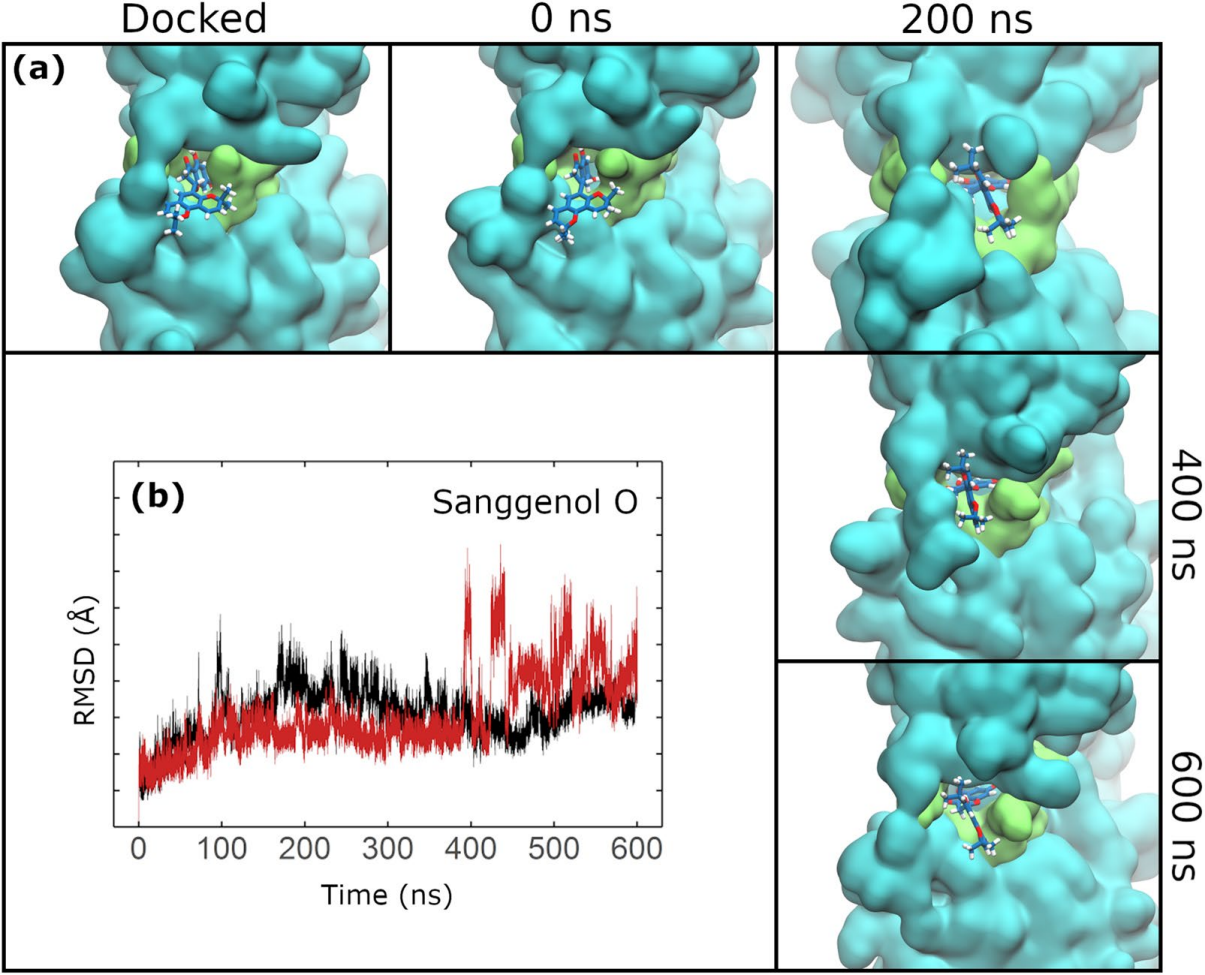
Sanggenol O-induced conformational changes in region A. (**a**) Snapshots of sanggenol O throughout the MD are shown each 200 ns. Residues from region A are shown in green surface while the rest of the protein is shown in cyan. Ligand backbone is coloured blue and other atoms follow the CPK colouring convention. (**b**) Ligand-induced RMSD variations in SBS residues are shown in red. RMSD of the *apo-*monomer structure is provided for comparison (black).

DS ligands sanggenol O (Fig. 4a) and CHEMBL2171573 (Supplementary Fig. 4), as well as CS ligand kurzichalcolactone (Supplementary Fig. 4), bound to region A during the whole MD. Sanggenol O caused constant fluctuations in protein RMSD (Fig. 3e). CHEMBL2171573 (Supplementary Fig. 5c) and kurzichalcolactone (Supplementary Fig. 5h) presented a similar behaviour. This phenomenon was likely due to constant modification of region A (Fig. 4a, Supplementary Fig. 4). From those ligands, only sanggenol O increased SBS RMSD around 390–410 ns (Fig. 4b). Additionally, CHEMBL2171584 initially bound to region A, but slowly migrated to domain III (data available upon request).

**Figure 5:**
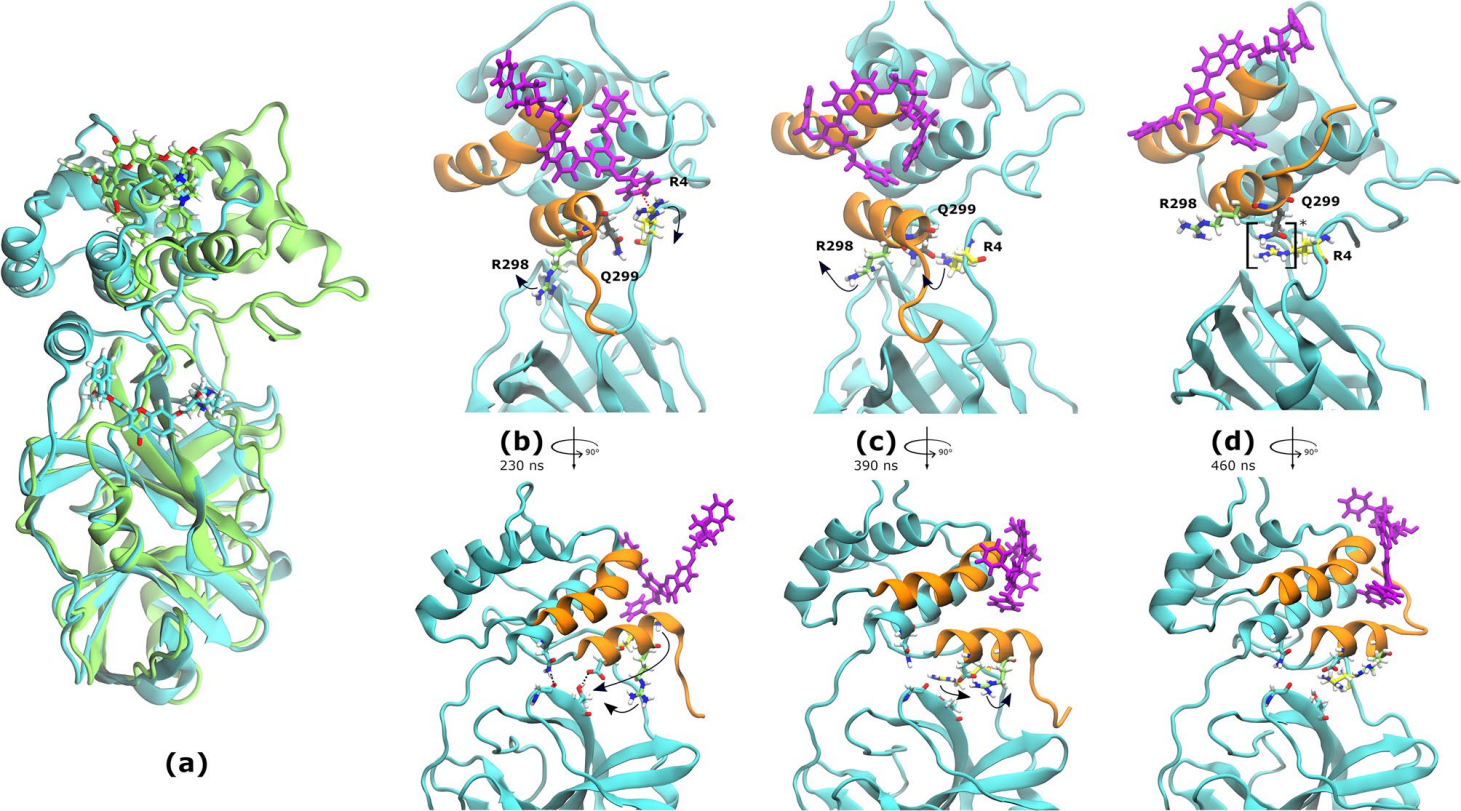
CHEMBL2171598 bound to region B and promoted the reorientation of M^PRO^ domains I/II. (**a**) Superimposition of the initial M^PRO^ complex (cyan; frame 0) to the 600 ns snapshot (green; frame 59,999) over the domain III. CHEMBL2171598 is shown as sticks. Movements of key residues are shown with black arrows. (**b–d**) MD snapshots detailing domain reorganization and the hinge mechanism. Key residues Arg4 (yellow), Arg298 (green) and Gln299 (gray) are displayed as sticks along with their interactions (red dotted lines for π-cation, black for electrostatic). Residues enclosed in [brackets]* are engaging in a polar contact. C-terminal helices (residues 244–257 and 293–306) that interact with the ligand are coloured orange. Lower panels are rotated 90° with respect to upper panels to display interactions between domains I/II and III. Residues participating in those interactions are shown as cyan sticks. Gln299 was removed for clarity. (**b**) CHEMBL2171598 induced the rotation of Arg4. Two polar contacts are observed between domains I/II and domain III: Gly109-Asn203 and Thr111-Asp295. (**c**) Arg4’s rotation reoriented Arg4 and Arg298 side chains in *syn* conformation. In their new *syn* conformation, Arg298 and Arg4 guanidinium groups interrupt the interdomain polar contacts. (**d**) Arg4 displaced Arg298 while CHEMBL2171598 adopted its final position, named Region B. Arg4’s insertion to the cleft made impossible for interdomain polar contacts to form again. The hinge movement was triggered shortly after, at 465 ns.

In CHEMBL2174598 simulations, an increase in protein RMSD was observed at 465 ns (Fig. 3f). The increase was due to a reorientation of domains I/II in relation to domain III (Fig. 5a). The movement resembled a hinge, and therefore it will be referred to as “hinge movement”.

The observed phenomenon correlated with ligand migration from region A to another protein section. During the process, CHEMBL2174598 entered in contact with Arg4 (Fig. 5b) and induced its rotation. At this point, two polar contacts were formed between domains I/II and domain III. The induced rotation oriented Arg4 and Arg298 side chains in *syn* conformation (Fig. 5c). Thus oriented, Arg298 and Arg4 guanidinium groups interrupt the interdomain polar contacts. By that time, CHEMBL2174598 had already adopted its final position, with a terminal aromatic ring inserted between helices conformed by residues 244–257 and 293–306. The final binding region will be named region B. Subsequently, at 460 ns, Arg4 inserted its side chain in the cleft between domains I/II and III, displacing Arg298 (Fig. 5d). Interdomain polar contacts did not form again, as they were now interrupted by Arg4 insertion to the cleft. Shortly after (at 465 ns), the hinge movement was triggered.

CHEMBL2171578 also triggered the hinge movement (Supplementary Fig. 6a). It attached to region B with a binding mode similar to that of CHEMBL2171598 (Supplementary Fig. 6b). After the landing, one aryl halide moiety pushed Phe8 (Supplementary Fig. 6c). Phe8 then displaced Arg298 and partially inserted its side chain into the I/II-III cleft (Supplementary Fig. 6d). However, CHEMBL2171578 was discarded for potential organochloride-mediated toxicity.

**Figure 6:**
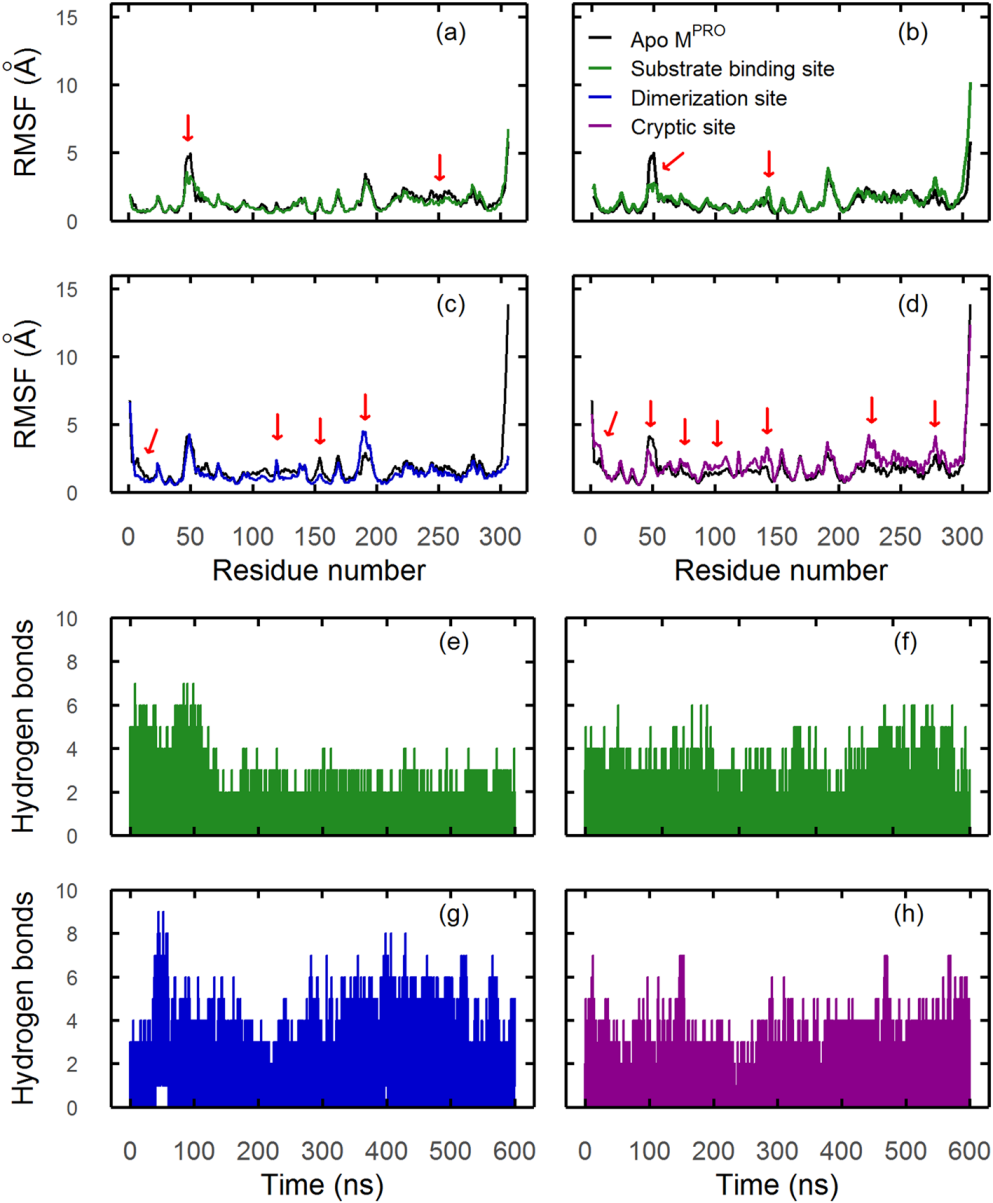
RMSF and hydrogen bond plots of flavonoid-SARS-CoV-2 M^PRO^ selected ligands. In both plot types, ligands are shown according to previous colour code: green for substrate, blue for dimeric and magenta for cryptic sites. (**a–d**) Protein RMSF plots. RMSF of *apo-* dimeric or monomeric (protomer A) protein is shown in black. Red arrows show the most significant differences between *apo-* and *holo-* structures. (**e–g**) Total number of hydrogen bonds formed between SARS-CoV-2 M^PRO^ and flavonoid ligands. (**a/e**)* Dorsilurin E* restrained the flexibility of residue 49. This ligand also had a long-range effect by reducing the flexibility of domain III residues 200–275. Dorsilurin E held the lowest average number of hydrogen bonds, 0.8 (s = 0.99). (**b/f**)* Euchrenone a11* restrained the flexibility of residue 49. Additionally, euchrenone a11 increased fluctuation in domain II (S1’ subpocket) residues 143–146. This ligand also held the second lowest average number of hydrogen bonds, 0.9 (s = 0.94). (**c/g**)* Sanggenol O* restrained the protein movement except for residues 186–198, which showed flexibilization. Sanggenol O shows remarkable fluctuations in its hydrogen bonds, holding an average of 1.6 hydrogen bonds through time (s = 1.30). (**d/h**)* CHEMBL2171598* increased the protein flexibility throughout its sequence, with a marked increase in domain II (residues 100–182) and residues 210–307 of domain III. CHEMBL2171598 held heterogenous hydrogen bonds through MD with an average of 0.9 (s = 1.01).

Regarding root-mean-square fluctuations (RMSFs), SBS ligand dorsilurin E (Fig. 6a) had a long-range effect on domain III by restraining residues 200–275. Additionally, euchrenone a11 (Fig. 6b) increased fluctuation in residues 143–146 from domain II (S1’ subpocket). DS ligand sanggenol O (Fig. 6c) significantly restrained the protein. A similar behaviour was observed for CHEMBL2171584 (Supplementary Fig. 7e). Finally, CS ligand CHEMBL2171598 (Fig. 6d) increased protein flexibility throughout its sequence, which corresponds with the large conformational change described before (Fig. 5a).

SBS ligands dorsilurin E (Fig. 6e) and euchrenone a11 (Fig. 6f) held an average of 0.8 (s = 0.99) and 0.9 (s = 0.94) hydrogen bonds through simulation time, respectively. That makes them the first and second most hydrophobic among selected SBS ligands. As for the DS, sanggenol O (Fig. 6g) showed remarkable fluctuations in its hydrogen bonds, holding an average of 1.6 hydrogen bonds through time (s = 1.29). Accordingly, sanggenol O is the most hydrophilic among selected DS ligands. CS ligand CHEMBL2171598 (Fig. 6h) held an average of 0.9 hydrogen bonds through time (s = 1.01) making it the most hydrophobic among the remaining selected CS ligands.

### MMPBSA

MMPBSA was computed by sampling three RMSD-stable states from the *holo* simulations. These states came from temperature, density and potential energy stable intervals, as such variables are practically constant during the 600 ns (Supplementary Figs. 8–10).

**Figure 7:**
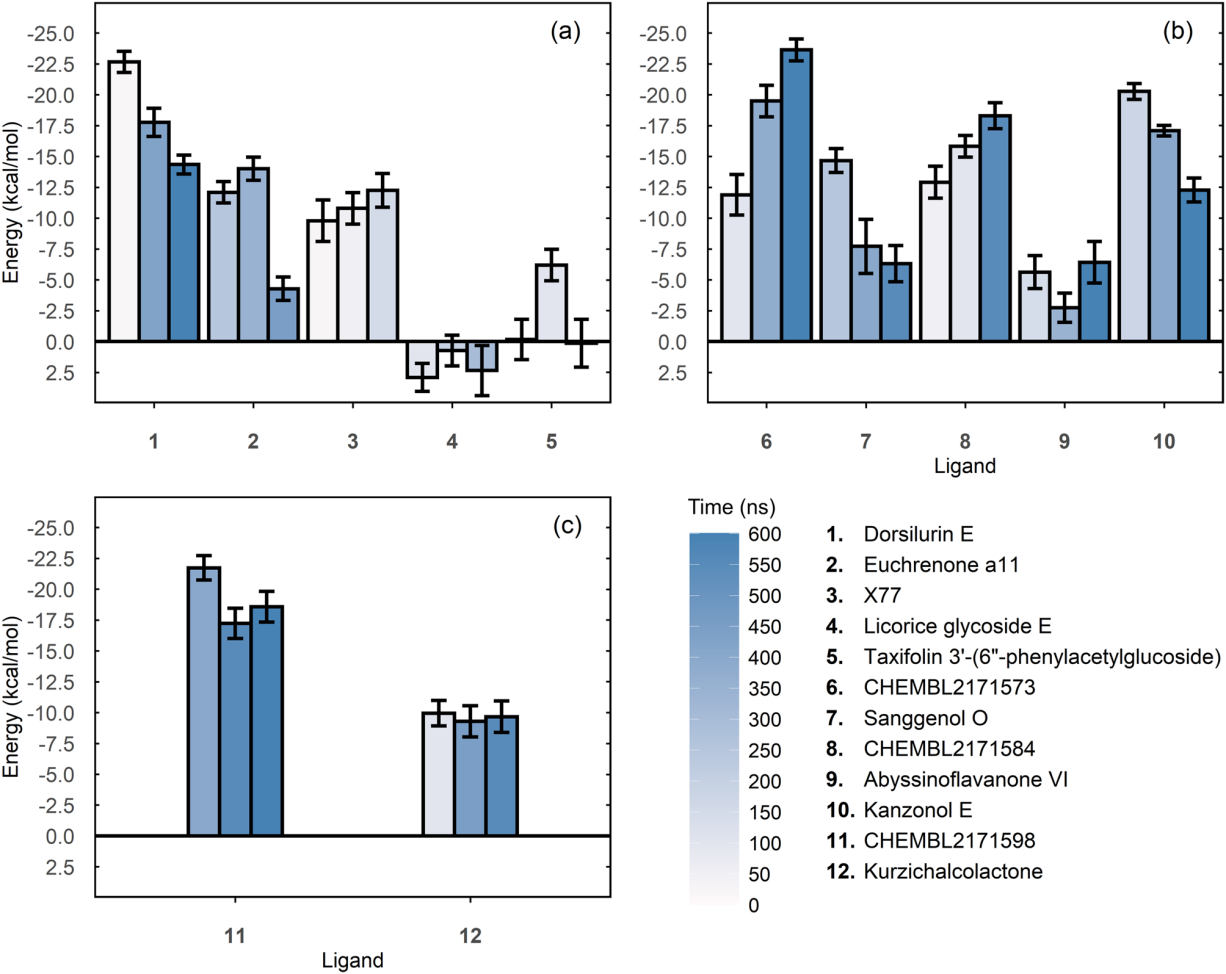
Mean and standard deviation values of binding energies calculated by the MMPBSA method. Mean binding energies are calculated from a N = 150 sample. Whiskers indicate standard deviations. Each N = 150 set was sampled by using a particular snapshot of the MD. Three snapshots were retrieved for each ligand. Darker blue filling indicates that snapshots have been taken from further along the holo MD. PBS: (**a**) SBS. Dorsilurin E had the most favourable energies, that started from − 22.66 kcal/mol (20 ns, s = 0.85) and gradually increased to − 4.34 (600 ns, s = 0.77). Its mean overall binding energy was − 8.25 kcal/mol. Positive control X77 had the second-best mean binding energy over time, with a peak at − 12.24 kcal/mol (150 ns, s = 1.37). Euchrenone a11 binding energies started from − 12.09 kcal/mol (250 ns, s = 0.88), decreased to − 14 kcal/mol (350 ns, s = 0.93) and then, closer to the unbinding event, stayed at − 4.27 kcal/mol (450 ns, s = 0.95). (**b**) DS. CHEMBL2171573 showed the lowest mean binding energy at − 18.33 kcal/mol, which decreased over time with a peak at − 23.63 kcal/mol (600 ns, s = 0.89). CHEMBL2171584 also showed decreasing mean binding energies over time that peaked at − 18.30 kcal/mol (550 ns, s = 1.04). Kanzonol E had an initial favourable value (− 20.26 kcal/mol, 175 ns, s = 0.64) that increased with time (− 12.27 kcal/mol, 600 ns, s = 0.97). This ligand also showed the least standard deviation values. (**c**) CS. CHEMBL2171598 started with a mean binding energy (− 21.72 kcal/mol, 400 ns, s = 0.98) that increased at 550 ns (− 17.23 kcal/mol, s = 1.22), only to decrease again at 600 ns (− 18.57 kcal/mol, s = 1.25). Overall, it showed the lowest mean binding energy at − 19.18 kcal/mol.

For the SBS (Fig. 7a), dorsilurin E had the most favourable energies. Its mean binding energy over the three snapshots was − 18.25 kcal/mol. Euchrenone a11 had an overall mean binding energy of − 10.1 kcal/mol, similar to X77’s (positive control). Among all DS ligands (Fig. 7b), CHEMBL2171573 showed the lowest overall mean binding energy at − 18.33 kcal/mol. Finally, CS ligand CHEMBL2171598 (Fig. 7c) showed the lowest overall mean binding energy of all binding sites at − 19.18 kcal/mol.

Based on the results described, a last filtering step was performed to select flavonoids for interaction analysis along MD (Supplementary Methods).

### Interaction analysis during ligand’s residence time

**Figure 8:**
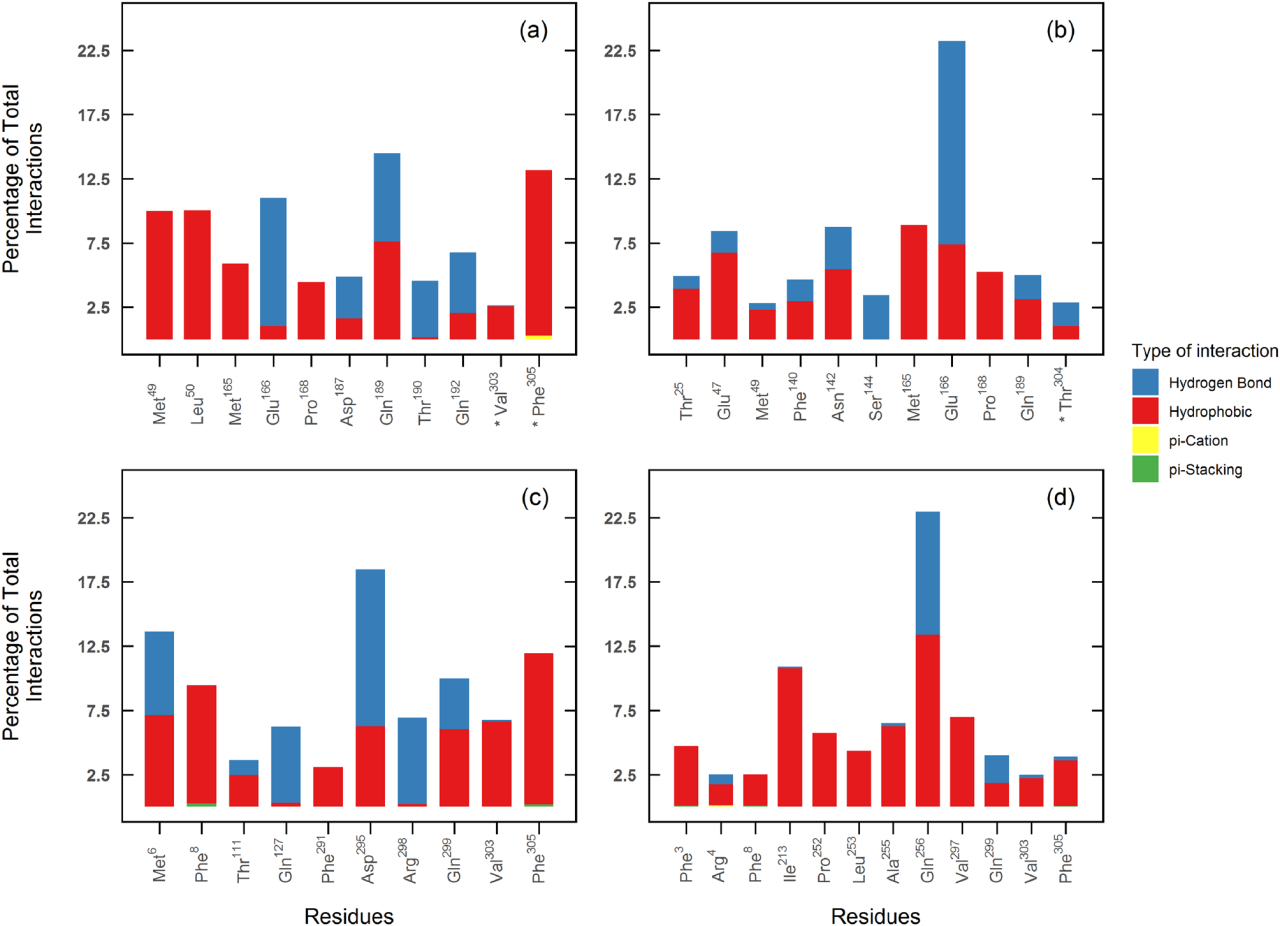
Interaction types for selected ligands as percentage of total interactions. Interaction types are limited to hydrogen bonds (blue), hydrophobic interactions (red), π-Cation contacts (yellow), and π-π stacking contacts (green). Residues with under 2.5% of interactions are not shown. Asterisks (*) indicate residues as belonging to protomer B, the default being protomer A. Note that not all ligands share the same number of interactions, so equal percentages do not mean equal number of interactions. *SBS*: (**a**) dorsilurin E, (**b**) euchrenone a11. *DS*: (**c**) sanggenol O. *CS*: (**d**) CHEMBL2171598.

Dorsilurin E (Fig. 8a) had a hydrophobic component of 65.91% in its interactions, higher than the 60.06% of euchrenone a11 (Fig. 8b). Interestingly, X77 (positive control) had a similar value (60.06%) (Supplementary Fig. 11a). The most persistent interactions held by dorsilurin E and euchrenone a11 were with Gln189 (14.46%) and Glu166 (23.22%) side chains, respectively. Notably, dorsilurin E and euchrenone a11 interacted with C-terminal residues from the other protomer. The contacts were more frequent for dorsilurin E.

For DS, sanggenol O (Fig. 8c) had the highest hydrogen bond percentage (40.67%). CHEMBL2171573 (Supplementary Fig. 11b) and CHEMBL2171584 (Supplementary Fig. 11c) had relatively high hydrophobic interaction percentages (83.67% and 85.16%, respectively), suggesting unspecific binding.

Finally, for CS, CHEMBL2171598 (Fig. 8d) exhibits the highest and second highest hydrogen bond percentages with Gln256 (9.62%) and Gln299 (2.17%), respectively. It also sustained interactions with the N-terminus where Arg4 enjoyed the most hydrogen bonds.

### Solvent-accessible surface area of SBS ligands along residence time

**Figure 9:**
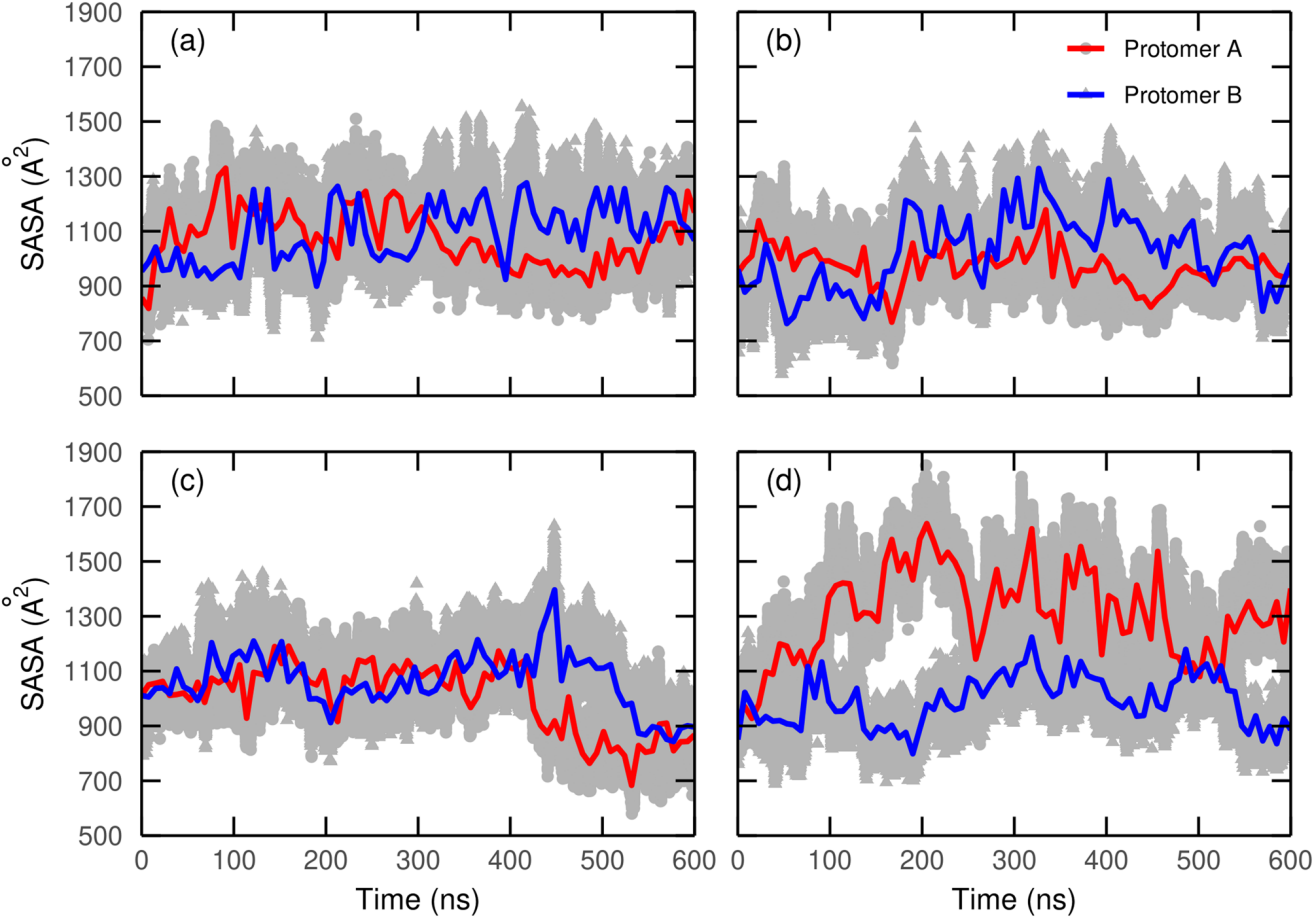
SASA values from SBS in each protomer along simulation time. SASA values correspond to protomer A and protomer B SBSs for (**a**) *apo* dimeric M^PRO^ or *holo* dimeric M^PRO^ with (**b**) euchrenone a11, (**c**) dorsilurin E or (**d**) X77 (positive control). Circles represent the real measure of SASA during simulation time for protomer A, while the same is true for triangles and protomer B. Lines are the smoothed values for each protomer (A, red; B, blue) that help in tendency recognition. Smoothing was done through locally estimated scatterplot smoothing (LOESS) using a span of 0.005. As was expected, X77 occupancy substantially increased overall SASA in protomer A SBS, thereby heightening the alternation pattern even without pronounced SBS closing in protomer B. Therefore, it is hypothesized that X77 would be exerting the same effect seen in the natural substrate and thus its main inhibition mechanism would not be the disruption of the alternation.

Solvent-accessible surface area (SASA) measurements were calculated for both M^PRO^ protomers in dorsilurin E, euchrenone a11 and X77 MD. For comparison, the same was computed for the *apo* dimeric M^PRO^ MD. In the *apo* M^PRO^ structure (Fig. 9a), an alternation of peaks and valleys among both protomers’ SBS regions was observed. Such pattern points at a SASA increase in one protomer’s SBS prompting a SASA decrease in the other. Euchrenone a11 (Fig. 9b) seemed to disrupt this alternation. An even more pronounced disruption was caused by dorsilurin E (Fig. 9c). Unlike the previous ligands, positive control X77 (Fig. 9d) increased the alternation pattern.

## Discussion

This study proposes dorsilurin E and euchrenone a11 as promising SBS inhibitors. Likewise, it suggests sanggenol O and CHEMBL2171598 as ligands with allosteric capabilities. This is the first time flavonoids have been proposed as allosteric inhibitors of M^PRO^.

SBS flavonoids reported here differ from others previously proposed^16,20–25,51^ for two main reasons. First, a previous lack of adequate PAINS filtering. Previous works proposed flavonoids that are confirmed to be PAINS (Supplementary Table 4)^27,52–54^, all of which were successfully filtered by the protocol applied here (Supplementary Dataset 1). Second, screenings starting from short libraries run the risk of presenting hits with unremarkable binding energies. For instance, mearnsitrin^20^ and pectolinarin^16^, two flavonoids previously proposed as inhibitors, were filtered in the fast-ranking screening because they had higher energies compared with the NSBS and ISBS top 100 (Supplementary Dataset 3a,b). Remarkably, dorsilurin E and euchrenone a11 had more favourable docking scores, supporting both as possible inhibitors.

During the screening process, sanggenol O and CHEMBL2171598 were within the top 30 in both the fast and the exhaustive-ranking stages (Supplementary Table 3). This consensus supports their inclusion as promising ligands. Consensus for dorsilurin E and euchrenone a11 could not be proven. It is important to remember that conformational search is higher in the exhaustive ranking. The reason why consensus was not found for SBS ligands might be because the rapid screening in Vina could not find the correct binding mode. This is further supported by the fact that binding modes for dorsilurin E and euchrenone a11 predicted during the fast and exhaustive ranking phases are different (data available upon request).

The exhaustive-ranking protocol followed here was validated. It allowed for the reconstruction of three experimentally determined complexes in the lowest energy binding mode (Fig. 2). RMSDs were low considering that the used M^PRO^ conformation was not the one originally bound to those ligands (cross-docking). Furthermore, the present protocol succeeded in filtering the negative control.

The *apo* MD showed that the RMSD of dimeric M^PRO^ stabilized before the monomeric one (Supplementary Fig. 3). SBS RMSD had less fluctuations in the dimer and SBS flexibility diminished, as RMSF for that region was lower. It seems that dimerization stabilizes the protein and its active site, which agrees with previous studies^9,10,12,13^. Interestingly, mass spectrometry assays did not find a substrate-monomer complex, either because the substrate cannot bind to a monomeric SBS or, if it does, it is a transient interaction^9^. This could be due to the higher flexibility of monomeric SBS observed here. As a similar situation could happen with ligands, drug screening in silico assays against SBS should be performed with a dimeric structure.

Despite its hydrophobicity, dorsilurin E was the best inhibitor for SBS as it had the best docking/MMPBSA energies and a RT of 100%. This may suggest that hydrophobic interactions have a favourable effect on ligand binding to SBS. The preference of SBS for hydrophobic ligands was also reported elsewhere^18,33^. Dorsilurin E is also the most polycyclic of the selected ligands, with 5 fused rings. Compounds with 3–7 fused rings reportedly had the best binding affinities with the protease^18^. Moreover, dorsilurin E caused a restraint in domain III residues 200–275 (Fig. 6a). Domain III is vital for dimerization in coronavirus main proteases^55^, so its restriction could hinder dimerization and hence catalytic activity. Furthermore, dorsilurin E had frequent hydrogen bonds with Glu166 during MD (Fig. 6e). This is a conserved residue^56,57^, which lowers the chance of resistant mutations.

Regarding euchrenone a11, protein RMSF showed flexibilization of residues 143–146 (within S1’ subpocket) (Fig. 6b). The destabilization could be linked with the formation of hydrogen bonds between this ligand and Glu166 during docking and MD (Supplementary Fig. 1, Fig. 8b). The protein–ligand interactions formed may have disrupted Glu166 contacts with other residues and caused the observed flexibilization. This presumption is logical because Glu166 is vital for SBS structure and function^8,13,57,58^. Glu166 stabilizes SBS by two hydrogen bonds that connect its carboxylic group with the amine and hydroxyl groups of the other protomer’s Ser1^34,58^. To confirm if these interprotomeric interactions are being disrupted, the distance between protomer A Glu166 Cγ (γ-carbon, AGlu166Cγ) and protomer B Ser1 Nε (ε-nitrogen, BSer1Nε) was computed. Distance between AGlu166Cγ and protomer B Ser1 Oγ (γ-oxygen, BSer1Oγ) was also calculated. In the *apo* MDs, distance averages of AGlu166Cγ···BSer1Nε and AGlu166Cγ···BSer1Oγ were 3.62 Å (s = 0.77) and 4.52 Å (s = 1.10), respectively. In the *holo* measurements, average distances were 5.46 Å (s = 3.60) and 6.60 Å (s = 3.75). Those differences suggest that interprotomeric interactions of Glu166 are being disrupted by euchrenone a11, which may explain SBS destabilization. The mechanism could impair M^PRO^ activity.

Surface analysis reaffirmed the alternation for both protomers in SBS opening (Fig. 9)^12^. This would require an allosteric communication between the SBSs of both protomers. Dorsilurin E disrupted the alternating behaviour of SBS. Euchrenone a11 also caused that behaviour effect but in less magnitude. This correlates with a marked interaction of dorsilurin E and the C-terminus of protomer B. It is possible then that C-terminus may be an allosteric communicator. However, more studies should be conducted to support this suggestion.

Sanggenol O bound to region A during the whole MD (Fig. 4). Coincidently, it diminished M^PRO^ flexibility on average (Fig. 6c). CHEMBL2171584 also bound to region A but slowly migrated to domain III. It also decreased protein flexibility (Supplementary Fig. 7e). It seems that binding to region A could have a negative impact on M^PRO^ flexibility. The active site of M^PRO^ is flexible and therefore its conformation is induced by ligand binding^8,12^. Hence, restraining the protein could affect M^PRO^ activity as its substrate would not be able to induce the SBS to its active orientation.

Sanggenol O was the only ligand to progressively enlarge region A. The same ligand caused a modification of the SBS which could inhibit natural substrate binding. Previous in vitro studies support the present observations, as they showed that binding to region A is possible^11^ and could inhibit M^PRO^ activity^9^. However, the principal explanation that these works give for the inhibition is dimerization disruption. Even though one study speculated that an allosteric mechanism should also be considered^9^, their data could not support such a proposition. Here, MD proved useful showing that binding to region A could have an allosteric effect over protein flexibility, apart from the immediate dimerization disruption.

CHEMBL2171598 bound to region B, inserting one aromatic ring in a hydrophobic cleft between helices conformed by residues 244–257 and 293–306 (Fig. 5c,d). This may lead to inhibition. Researchers have proven that compounds that bind to the cleft inhibit virus replication^10^. Additionally, CHEMBL2171598 triggered a hinge movement between domains I/II and III (Fig. 5a). Surprisingly, the hinge movement was experimentally observed when Arg298 was mutated to alanine in the SARS-CoV M^PRO^^55^. The mutated protomer lost dimerization and catalytic activity. Therefore, CHEMBL2171598 supposes a promising ligand that could impair M^PRO^ by emulating the effect of the deleterious mutation Arg298Ala.

We propose a possible mechanism as useful in future works that try to trigger the hinge movement with ligands. (1) CHEMBL2171598 interacted with N-terminal residue Arg4 and induced a rotation of the residue (Fig. 5b). (2) The rotation slowed down when Arg4 and Arg298 side chains were in a *syn* conformation, because of electrostatic and steric strain (Fig. 5c). Arg298 acted as a gate that opposed resistance to the rotation of Arg4. (3) A favourable electrostatic contact was formed between the side chains of Arg4 and Gln299 (Fig. 5c,d). That might have exerted an opposing force to the resistance of Arg298, allowing the rotation to continue. (4) Arg298 was pushed by the repulsion of Arg4 guanidinium and the displacement of the C-terminal helix exerted by CHEMBL2171598 aromatic ring. This finished the gate-like opening of Arg298 and allowed Arg4 access to the I/II-III interdomain cleft (Fig. 5d). (5) Insertion of Arg4 might have caused steric strain on the atoms inside the cleft. Considering that Arg298 was severely displaced, the steric strain may have been sufficient to trigger the hinge movement.

In the SARS-CoV mutated structure (PDB ID: 2QCY)^55^, Lys5 side chain inserted into the cleft between domains I/II and III. These findings support the proposal of Arg298 as a gate to the interdomain cleft. As the mutated structure does not have the bulky guanidinium group at position 298, the entrance of Lys5 might be allowed. Although the importance of Arg298 has been recognised previously^55^, its gate-like behaviour for the interdomain cleft is novel.

Arg298 is compromised by either displacement or deletion in the dynamics of CHEMBL2171598, organochloride CHEMBL2171578 and in the mutated SARS-CoV M^PRO^^55^. All three cases allowed for an N-terminal residue (Arg4, Phe8 or Lys5, respectively) to enter the cleft. Collectively, this all points towards a general gate-like mechanism, in which displacement of gate Arg298 and insertion of N-terminal residues into the interdomain cleft cause the hinge movement.

600 ns of MD were sufficient to observe the promising allosteric effects of sanggenol O and CHEMBL2171598, which occurred after 400 ns. This is coherent with previous studies suggesting 250 to 500 ns of simulation time to study allosterism^59,60^. The present simulations spot flexibility differences between *apo* monomeric and dimeric M^PRO^. Hence, it was concluded that 600 ns were sufficient to address the conformational dynamics of the M^PRO^ in the *apo* and *holo* cases.

A possible methodological limitation is the use of the CGenFF program. The mentioned software parametrizes ligands by analogy. Therefore, the reliability of the parameters will depend on the quality of the analogy. Although previous studies in flavonoids have successfully used CGenFF^61–63^, forces may be more accurately estimated by quantum mechanics methods^64–66^. However, the approach would increase computation time, deviating from the rapid screening strategy that may be more valuable given the current global emergency. Nonetheless, even if parametrization can be improved, ligands can aid in the prediction of structural inhibitory mechanisms within the protein because protein force fields are reliable. Suboptimal parametrization can still produce ligands that act as perturbation agents to scan the conformational response of the protein^67^. Therefore, structural mechanisms proposed here are useful for future drug design and inhibition quests.

In summary, dorsilurin E is reported as a promising inhibitor of SBS. Euchrenone a11 also supposes a worthy candidate. Both interacted with important M^PRO^ residues and exerted structural effects coherent with the literature. Moreover, they disrupted the alternating induced-fit mechanism proposed for M^PRO^. Targeting DS and CS permitted to identify sanggenol O and CHEMBL2171598 as possible allosteric inhibitors, binding regions A and B, respectively. It allowed the observation of a novel allosteric effect in M^PRO^ flexibility when ligands bind to region A. MD allowed to propose a new allosteric mechanism related to the binding to region B, which caused a hinge movement between domains. The present data suggest that triggering that movement with a ligand to inhibit M^PRO^ is a possible and striking strategy. The structural mechanism described here will serve as a basis for future works that aim to trigger the hinge movement with new ligands.

## Methods

### Library preparation

An exhaustive web scraping using the *rvest* package^68^ was conducted for compounds reported as flavonoids in the database of Arita Laboratory (National Institute of Genetics, Japan)^69^. Similarly, the online databases DrugBank^70^, CHEMBL^71^ and PubChem^72^ were subjected to automated search. For these last websites, searches were performed manually using key words “flavonoid”, “flavonoids” and “flavo”. All four bases were standardized to show a unique ID, SMILE and online source for each compound before merging. Compounds with molar weights under 180 g/mol and repeated flavonoids (with identical SMILES) were removed.

### In silico PAINS filtering

To control for false positive findings during the high throughput screening, pan-assay interference compounds were detected. The complete database was then uploaded to FAFDrugs4 server^73^ for further processing. Two SMILEs, even though literally distinct, may be synonymous. FAFDrugs4 reads the SMILEs into a structure to exclude synonymic ones. Next, all screening tools to identify and purge PAINS offered by the website were employed. A final SMILE database was downloaded and matched for their unique IDs and online sources.

### 3D structure generation

OpenBabel v.3.1.1^74^ was used to interpret SMILE files into bidimensional structures. These in turn were translated into three-dimensional structures and subjected to an energy minimization through the steepest-descent method^75^ using the same software. The minimization used the MMFF94 forcefield^76^ with a convergence criterion of 1 × 10^–7^ kcal/mol. All these steps were performed automatically through an in-house Python script.

### Ligand preparation

Twenty compounds were randomly selected for manual quality control of structure generation. All torsions were assigned and Gasteiger charges were added for all atoms in the compound structures using AutodockTools v.4.2 package^77^. Flavonoids with more than 32 torsions were filtered out.

### Receptor preparation

Two distinct M^PRO^ conformations were used: a ligand-free conformation (PDB ID: 6YB7)^45^ and another conformation induced by an inhibiting ligand (PDB ID: 6LU7)^34^. These structures were independently curated on PDBfixer^78^ with the intent of repairing lost atoms and residues, as well as for removing water molecules and other heteroatoms. It is necessary to perform SBS screening against the dimeric conformation of M^PRO^ given the recent evidence of the interplay between both protomers^8,12^. DS and CS location requires methodology based on monomeric M^PRO^. Therefore, receptors were constructed using these repaired chains as monomers as well as dimers from the corresponding biological assembly available at Protein Data Bank. Polar hydrogens and Gasteiger charges were added to all receptor structures using AutodockTools v.4.2 package^77^.

### Definition of putative binding sites

First, three PBSs were identified for M^PRO^ docking: SBS, DS and CS. The first two were chosen after bibliographic consideration^7,11,13,34–36^ while CS was originally predicted using CryptoSite^14^. Briefly, CryptoSite produced a file containing scores for every residue. Residues with scores higher than 10 were selected, as they have a high probability of being part of a cryptic site. From this initial selection, several residues were within the same region of the SBS or DS. These were left out for the sake of dynamic independence.

A cross-docking methodology was followed to reach robust conclusions for SBS screenings. The ligand-free M^PRO^ (PDB ID: 6YB7)^45^ and the ligand-induced M^PRO^ (PDB ID: 6LU7)^34^ prepared before were the receptors, both in dimeric form. The former’s protomer A SBS and the latter’s protomer A SBS are referred to as NSBS and ISBS, respectively. A dimeric assembly is preferable because the SBS of one protomer is near the N-terminus of the other^8,12^. This limits SASA available for binding. Additionally, amino acids of the other protomer could contribute to ligand binding. Hence, docking against a monomeric MPRO could cause miscalculations in SBS screenings.

For DS and CS, the unligated M^PRO^ (PDB ID: 6YB7)^45^ in its monomeric form was the chosen receptor, because using a conformation with a ligand could affect the geometry of DS and CS. Inhibition against these sites should be tested with a protein in its natural, ligand-free conformation.

Among the crystals with similar conformations available, PDB ID: 6YB7^45^ and PDB ID: 6LU7^34^ were those with the best resolution. Conveniently, both were of similar quality to allow comparison.

For every screening procedure, the search box size and coordinates were optimized to include the Van der Waals volume of all residues comprising each site. Dimensions and coordinates of NSBS, ISBS, DS and CS search boxes are presented in Supplementary Table 5.

### Fast-ranking screening

VS pipelines for medium or large-datasets usually consist of two phases: an initial, fast-ranking screening followed by an exhaustive-ranking run^33,37–41^. The present flavonoid library of 4858 flavonoids was large enough to follow a two-phase screening^33,38,40^. Hence, a two-step methodology was applied.

Vina^42^ was chosen for the fast-ranking screening. There, a semiflexible VS (with rigid receptor and flexible ligand) was conducted with 24 exhaustiveness, as recommended elsewhere^79^. For each docking, the lowest energy binding mode was selected.

The selection of Vina for the first ranking stage is due to its efficiency^42^. As it is a fast algorithm, Vina is recommended for an initial fast screening of numerous molecules^80^. Multiple studies have used Vina for fast-ranking phases^33,39–41^.

### Exhaustive-ranking run

The top 100 compounds of the fast-ranking stage were re-evaluated through a more exhaustive run on AutoDock-GPU^46^. Coordinates produced in this stage were the ones chosen for analysis.

A Lamarckian genetic algorithm (LGA)^81^ was used as a global search method. The local search was directed after the Solis-Wets algorithm (SWA)^82^. The procedure included 25 million evaluations and 150 runs, with a box spacing of 0.375 Å. In each exhaustive docking assay, the best pose was selected based on its ${2D}_{score}$^47^. Briefly, each pose is assigned a score based on the standard normalization (${Z}_{score}$) of two variables: binding energy (ΔG) and cluster population (*Pop*).

1 $${2D}_{score} = -1\times [{Z}_{score}(\Delta G)]+{Z}_{score}(Pop)$$

The solutions were combined and ranked according to their binding energies. The top 30 ligands with the lowest binding energies against NSBS and ISBS were compared. The ones present in both lists were selected as ligands with probability to bind the SBS. In the case of DS and CS, the top 5 ligands with lowest binding energies were chosen.

The most favourable poses of the selected ligands were extracted and supplemented with the missing hydrogen atoms using OpenBabel at pH 7.4. Hydrogens for the receptor were supplied with PDB2PQR^83^ at the same physiological pH. The protein–ligand complexes were reconstructed by merging the ligand files with their corresponding hydrogen-curated receptor for further analysis. It is worth noting that for SBS, just ISBS complexes were built (using dimeric receptor PDB ID: 6LU7^34^) because of its binding energies being more favourable than the ones of the NSBS.

The reason why AutoDock-GPU was selected for the second phase is because, besides binding energy, it provides the cluster population of the binding mode. The two parameters can be combined into the 2Dscore to quantitatively select the most probable binding mode. Moreover, for an exhaustive-ranking stage, it is necessary to adjust multiple parameters to obtain the best results. For instance, AutoDock-GPU allows users to select the best population size and number of evaluations. This multiple-parameter tweaking cannot be done with Vina.

The specific approach to employ AutoDock4.2 to validate Vina results is supported by multiple works^33,39–41^. Similarly, AutoDock4.2 has been used to validate results from Glide^38^.

### Positive and negative controls

It is key to confirm that the coordinates produced in the exhaustive-ranking stage replicate the binding modes of known experimental inhibitors (positive controls). Therefore, non-covalent M^PRO^ inhibitors X77, X7V and YD1, were chosen. The three ligands were excised from their co-crystalized complexes (PDB IDs: 6W63^48^, 7KX5^49^ and 7LTJ^50^, respectively). Ligands were manually curated to correct bond order and add missing hydrogen atoms. The three controls were then docked against ISBS with the same parameters of the exhaustive-ranking stage. The docked protein–ligand complex was built following the procedure detailed before. The resulting complexes were compared with their experimentally determined conformations (PDB IDs: 6W63^48^, 7KX5^49^ and 7LTJ^50^) by RMSD.

Equally important is the capacity of sieving protocols for rejecting inert molecules. These compounds could exhibit false positives in biochemical assays due to colloidal aggregation^84,85^. For example, TDZD-8 only inhibits M^PRO^ through aggregation^34^. Thus, TDZD-8 was taken as a negative control to test the efficacy of the present protocol at excluding promiscuous molecules. Docking and complex preparation of TDZD-8 followed a similar protocol to that of the positive control.

### Docking interaction analysis

Protein–Ligand Interaction Profiler (PLIP) v.2.1.6^86^ was used with default parameters to further explore protein–ligand interactions arising from the docking. Regarding the SBS, it is worth mentioning that only the interactions provided by the exhaustive-ranking stage against ISBS were identified and analysed.

### Molecular dynamics

The protein–ligand complexes reconstructed before, as well as positive (X77) and negative (TDZD-8) controls, were subject to MD simulations with NAMD 2.13^87^. CHARMM36^88^ was the selected forcefield. The ligands’ parameterization was generated with the CHARMM General Force Field (CGenFF) program v.2.2.0^89^.

For each complex, NaCl counterions were placed in the protein’s surface based on its coulombic potential using the CIonize 2.0 package^90^ from VMD^91^. Counterions placed farther than 20 Å were eliminated. After that, protein–ligand complexes were solvated in an optimized cubic box based on the size of the complex. 15 Å of padding was used. Salt concentration was set to 0.154 M (physiological concentration) with AutoIonize v.1.5 package developed by Ilya Balabin.

First, only water atoms were energy-minimized by 15,000 steps using the conjugate gradient algorithm. Then, MD of the same atoms at 0 K for 0.03 ns were conducted. The whole system was then stabilized by 50,000 steps of energy minimization (conjugate gradient algorithm), until energy converged at around − 379,656.5 kcal/mol for dimeric systems and − 638,224.4 kcal/mol for monomeric systems. To allow comparison, it was considered appropriate that dimeric and monomeric systems MD started from states with similar energies. 50,000 steps were necessary to reach differences in energy of less than 6% at the last minimization step. Specifically, all dimeric systems differed in energy by less than 1%, while monomeric ones differed by less than 6%.

Equilibration of temperature and pressure were addressed by following the procedure described by Hadden and Perilla^92^. Both the Langevin thermostat and Noose-Hoover Langevin barostat were used in the NPT ensemble. First, the complex was heated from 50 to 310 K at a rate of 5 K per 0.01 ns. For this, harmonic restraints were applied to the protein’s backbone and all ligand atoms (except hydrogens in both cases) with a force constant of 5 kcal/mol. Then, the forces on the system were reduced by 10% each 0.05 ns. Finally, 100 ns of unrestrained MD of the NPT ensemble were performed. If the ligand remained associated with the protein during the 100 ns, the unrestrained MD was extended 500 ns. The purpose was to evaluate possible conformational changes as well as to evaluate RT. Velocity was restarted for these extensions according to the Maxwell–Boltzmann distribution to augment conformational sampling.

All the processes described were performed in periodic boundary conditions with an integration time of 2 fs/time-step and Particle Mesh Ewald (PME) grid spacing of 1.0 Å. Cutt-off for non-bonded interactions was set to 12 Å. In addition, *apo* protease MD was performed both in its monomeric and dimeric conformations following the same procedure. As with all SBS MDs, these were carried out with the protease in its dimeric form prepared from the hydrogen-curated crystal PDB ID: 6LU7^34^.

### MMPBSA

Results obtained from docking can derive in false positives^32,93,94^. MD approaches to re-score the docking results could help to reduce the frequency of these misreports. One of these methods is the MMPBSA protocol, which states that Gibbs energy related to a structure can be decomposed as follows:

2 $${\langle G\rangle }_{i}={\langle {E}_{total}+{G}_{sol}-TS\rangle }_{i}$$

where *E*_*total*_ is the energy associated with molecular dynamics. *G*_*sol*_ is the solvation free energy. Brackets indicate ensemble averages computed from the simulation *i*.

In the “single-trajectory” MMPBSA approach, binding energy is denoted by:

3 $$\Delta {G}_{bind}={\langle {G}_{PL} - {G}_{P}-{G}_{L}\rangle }_{PL}$$

*PL*, *P* and *L* stand for protein–ligand, protein, and ligand, respectively. Note that (3) is an ensemble average over the protein–ligand simulation. Replacing (2) in (3):

4 $$\Delta {G}_{bind}={\langle \Delta {E}_{PL}^{int}\rangle }_{PL}+{\langle \Delta {G}_{PL}^{sol}\rangle }_{PL}-{T\langle \Delta {S}_{PL}\rangle }_{PL}$$

where ${\langle \Delta {E}_{PL}^{int}\rangle }_{PL}$ is the protein–ligand interaction energy from MD, ${\langle \Delta {G}_{PL}^{sol}\rangle }_{PL}$ is the change in the solvation energy upon binding and ${T\langle \Delta {S}_{PL}\rangle }_{PL}$ is the protein–ligand interaction entropy multiplied by the absolute temperature T in kelvin.

$\Delta {G}_{bind}$ calculation was computed from (4) ignoring the entropic term. To this end, the software CaFE v.2.0^95^ was used. Temperature was set at 310 K. For the calculations of solvation energies, the water (exterior) dielectric constant at 310 K was estimated by a polynomial fit^96,97^ and set to 74.2.

Note that Eq. (4) gives the $\Delta {G}_{bind}$ of one protein–ligand MD. More MD replicas should be generated to diminish the standard error^98^. Therefore, for each protein-drug complex (including the positive control), three snapshots were retrieved from different RMSD-stable states. Temperature, potential energy and density were examined before selecting the snapshots to see if they reached convergence. 150 replicas were run for 0.25 ns using each snapshot as starting point, assigning different velocities to each replica based on the Maxwell–Boltzmann distribution at 310 K. Free binding energy of each replica was calculated using Eq. (4) (ignoring the entropic term), after which an arithmetic average over the 150-set was given.

### RMSD and RMSF

RMSDs of all frames of protein–ligand trajectories were calculated with an in-house Tcl script using VMD built-in functions and the module “bigdcd”. Frame 0 was used as the reference to which all subsequent frames were aligned. Ligand RMSDs were computed using all non-hydrogen atoms while protein RMSDs were from the alpha carbons. As for the RMSF calculation, the trajectory was loaded with a step of 5, aligned using frame 0 as a reference and the RMSF computed in the modified coordinates. RMSFs of protein–ligand complexes that remained for at least the first 100 ns were measured and compared with the RMSF of the corresponding *apo* structure (*apo* dimeric for SBS and *apo* monomeric for CS and DS complexes).

### Interaction analysis along ligand’s residence time

The intervals where the selected ligands remained bound to M^PRO^ for each trajectory were converted to independent files. In these files, each conformation was equivalent to a single frame of the interval. Then, the independent files were used as input for an in-house pipeline that employs Python and Bash programming languages. Briefly, the pipeline determines the ligands’ interaction types at the PBS for each frame through the PLIP v.2.1.6 software^86^. Finally, recount and reordering according to the interaction type is performed for each residue to draw a bar plot. For this analysis, a 2.5% cut-off is set to mark the main interacting residues for each compound.

### Solvent accessible surface area of SBS ligands

Previous authors have noted that protomers A and B are dynamically different^12^. They observed that when the SBS volume of protomer A increased, the volume of protomer B decreased, and inversely. With such an observation, the authors proposed an induced-fit mechanism that governs the behaviour of one protomer when the other is bound to a substrate. To evaluate such observations when working with inhibitors as substrates, SBS SASA of both protomers were analysed in the trajectories of SBS ligands selected for interaction analysis. To have a comparison, this protocol was also applied for the *apo* dimeric MD. SASA was computed using an in-house Tcl script in a similar manner as applied for obtaining the RMSDs.

### Figure preparation

Figures 1, 2, 4a and 5, as well as Supplementary Figs. 1, 2, 4a and 6 were rendered using VMD 1.9.3 software^91^. Panels were assembled using GIMP v.2.10.18 (https://www.gimp.org/downloads/). In Fig. 5 and Supplementary Fig. 6, arrows were drawn using Inkscape v. 1.0.2–2 (https://inkscape.org/). Figures 3, 4b, 6, 7, 8, and 9, as well as Supplementary Figs. 3, 4b, 5 and 7–12 were generated using R v.4.0.1 (https://cran.r-project.org/) in RStudio 1.1463 environment (https://www.rstudio.com/products/rstudio/download/).

### Preprint

A previous version of this manuscript has been deposited on a preprint server: https://arxiv.org/abs/2008.13264.

## Supplementary Information

Supplementary Information 1.

Supplementary Dataset 1.

Supplementary Dataset 2.

Supplementary Dataset 3.

Supplementary Dataset 4.

## Data Availability

All relevant data are contained within the manuscript and the Supplementary Material. Additional raw data will be available upon request.
