# Supplementary material for: Comprehensive virtual screening of 4.8 k flavonoids reveals novel insights into allosteric inhibition of SARS-CoV-2 MPRO
Source: Sci Rep. 2021 Jul 29;11:15452. doi: 10.1038/s41598-021-94951-6 (PMC8322093; doi:10.1038/s41598-021-94951-6)
Supplement: Supplementary file 1 — Supplementary Information 1. [file 41598_2021_94951_MOESM1_ESM.pdf]

SUPPLEMENTARY MATERIAL

Comprehensive virtual screening of 4.8k flavonoids reveals novel insights into allosteric inhibition of SARS-CoV-2 M^{PRO}

Gabriel Jiménez-Avalos[1]§*, A Paula Vargas-Ruiz[1]§, Nicolás E Delgado-Pease[1]§, Gustavo E Olivos-Ramirez[1], Patricia Sheen[1], Manolo Fernández-Díaz[2], Miguel Quiliano[3], Mirko Zimic[1,2]*, COVID-19 Working Group in Perú.

AFFILIATIONS:

1. Laboratorio de Bioinformática, Biología Molecular y Desarrollos Tecnológicos, Facultad de Ciencias y Filosofía, Departamento de Ciencias Celulares y Moleculares, Universidad Peruana Cayetano Heredia (UPCH), 15102, Lima, Perú.

2. Farmacológicos Veterinarios - FARVET S.A.C. Chincha, Perú.

3. Faculty of Health Sciences, Centre for Research and Innovation, Universidad Peruana de Ciencias Aplicadas (UPC), 15023, Lima, Peru.

§ Equally contributing authors.

*Corresponding authors: gabriel.jimenez.a@upch.pe, mirko.zimic@upch.pe

A previous version of this manuscript has been deposited on a preprint server: https://arxiv.org/abs/2008.13264

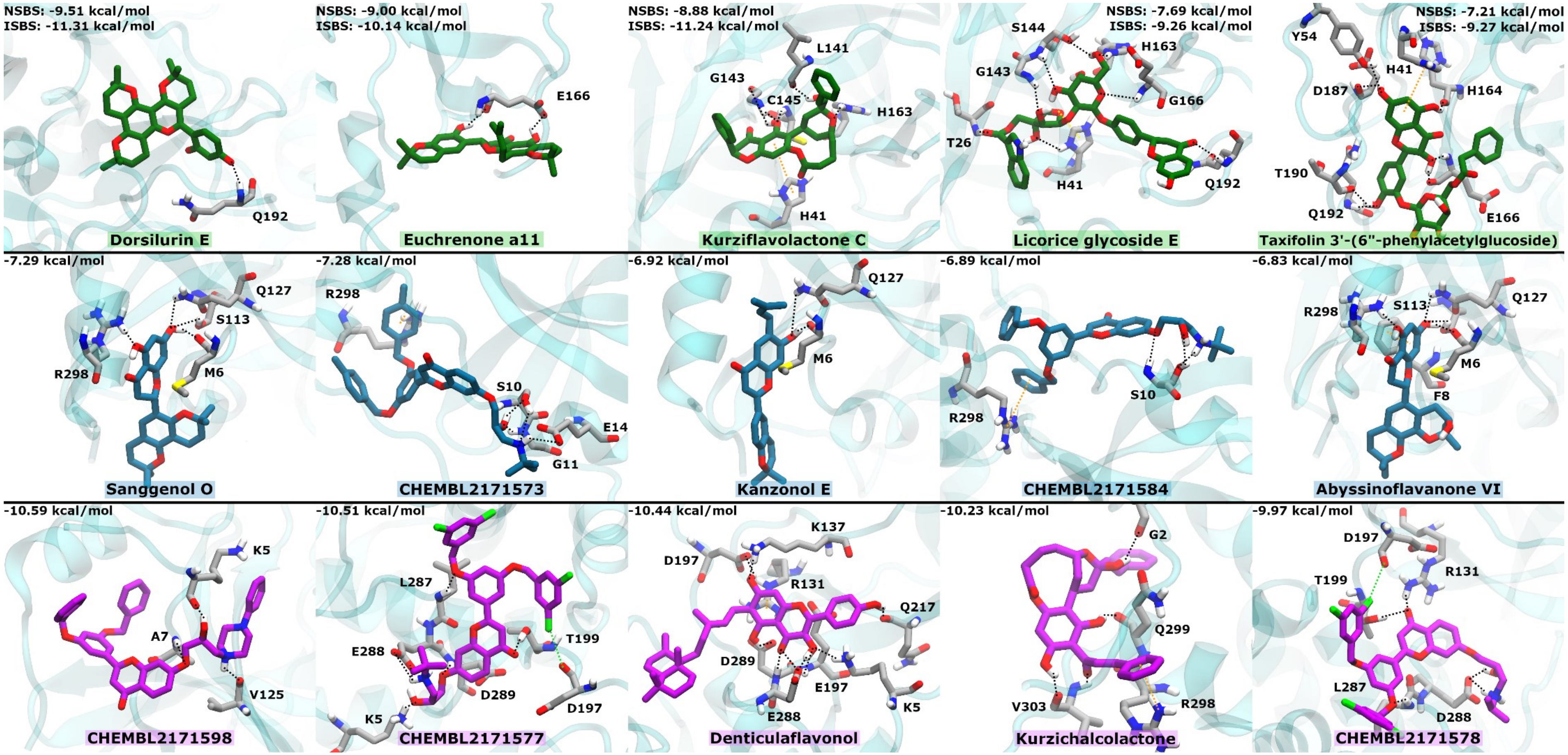

Supplementary Figure 1. Interactions of the top 5 binding energy compounds. PBS are in order of appearance and ligands are colour-coded: induced-fit substrate binding site (1° row, green), dimerization site (2° row, blue) and cryptic site (3° row, magenta). Both ligands and interacting residues (silver) are shown as sticks. Non-carbon atoms are coloured following the CPK colouring convention. Protein is depicted as cartoon. The common name of each ligand is shown, except for those where only a code is available. Main interactions are shown as dotted points as follows: hydrogen bonds in black, π-π or π-cation interactions in orange and halogen bonds in green. All DS ligands inserted at least one of their aromatic rings into a small pocket. The buried rings of CHEMBL2171573 and CHEMBL2171584 sustained a π-cation interaction with Arg298 (Figure 2, Supplementary Table 2). Abyssinoflavanone VI showed a π-π stacking with Phe8. The buried rings of abyssinoflavanone VI, sanggenol O and kanzonol E (FL3F1ANP0001) are substituted with hydroxyl groups. In both abyssinoflavanone VI and sanggenol O, the hydroxyl group in the position 5 of the backbone ring shows hydrogen bonds with Arg298. Likewise, in both flavonoids the hydroxyl group in position 7 presents three hydrogen bonds with Met6, Ser113 and Gln127. Furthermore, the hydroxyl group in the position 7 of the kanzonol E ring established hydrogen bonds with Met6 and Gln127. Kurzichalcolactone and CHEMBL2171598 were found to bind within region A in the same fashion as the DS ligands.

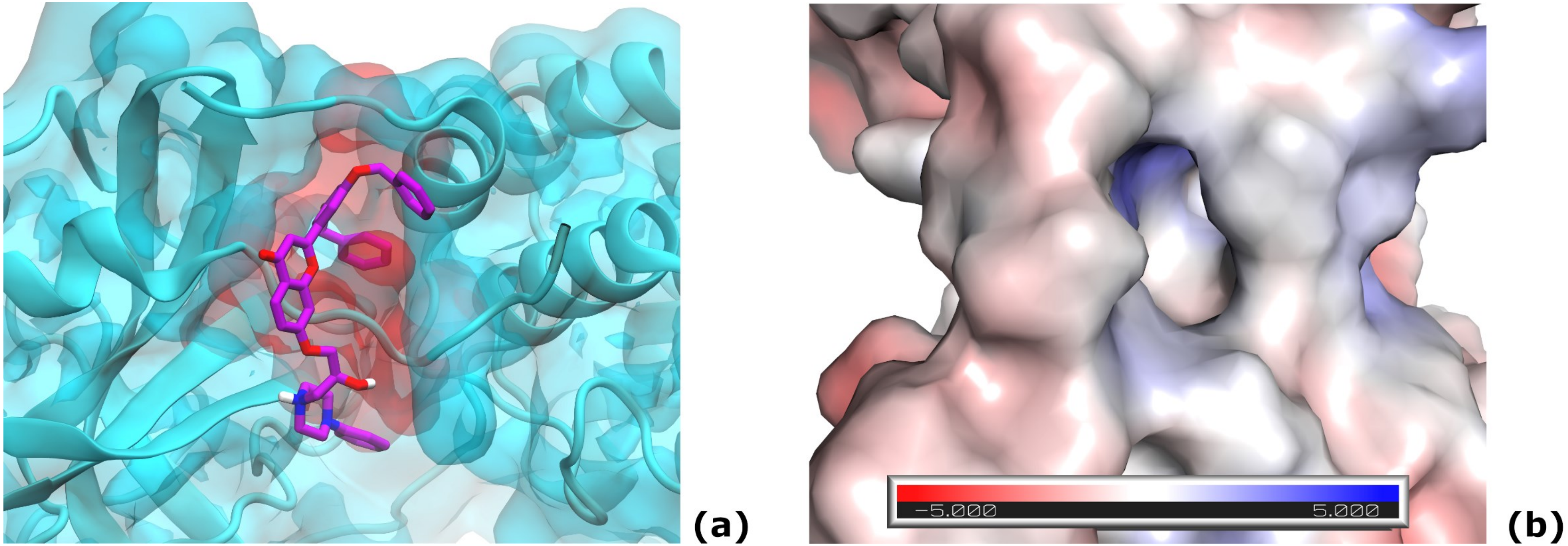

Supplementary Figure 2. Close-up and electrostatic potential surface of Region A. Region A is composed by the following residues: Lys5, Met6, Ala7, Phe8, Ser113, Gln127, Glu290, Phe291, Asp295, Arg298, Val303. **(a)** A representative compound from those tested by docking assays against DS and CS, CHEMBL2171598, is shown in magenta sticks as it slides an aromatic ring into the novel cavity. The surface of residues comprising region A is shown in red, whereas the rest of the protein is in cyan. **(b)** Electrostatic potential surface for region A's pocket. Electrostatics calculations showed that region A's pocket has a predominantly neutral surface, with exception of some moderate positively charged areas that coincide with the side chains of Lys5, Gln127 and Arg298. Also, slightly negatively charged fragments coherent with the side chain of Asp295 were noticed.

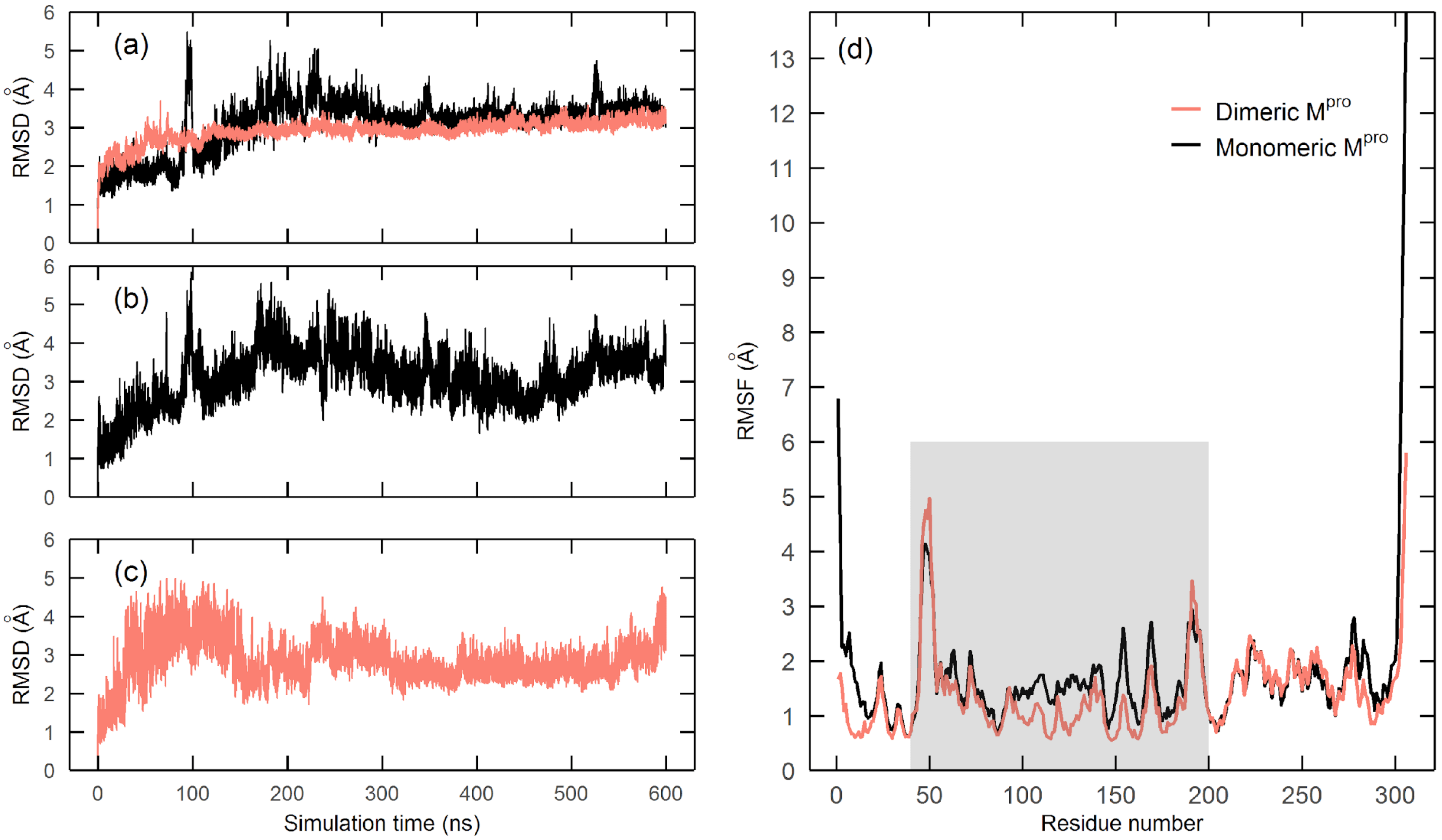

Supplementary Figure 3. RMSD and RMSF plots of free SARS-CoV-2 M^{PRO} (a) RMSD fluctuation of free M^{PRO}. **(b)** RMSD fluctuation of SBS residues in the monomeric M^{PRO}. **(c)** RMSD fluctuation of the SBS residues of dimeric M^{PRO} protomer A. **(d)** RMSF fluctuation of free M^{PRO}. Gray area highlights an area that virtually include all residues from the SBS (Ser1*, His41, Met49, Tyr54, Phe140, Leu141, Asn142, Gly143, Ser144, Cys145, His163, Met165, Glu166, Leu167, Pro168, His172, Phe185, Asp187, Gln189, Thr190, Ala191, and Gln192). Asterisks (*) indicate residues as belonging to protomer B, the default being protomer A. Data from dimeric M^{PRO} is depicted in pink whereas that of monomeric M^{PRO} is in black (PDB: 6LU7)[1]. For the dimeric conformation, data was calculated from only one chain. Molecular dynamics time is shown in 100 ns intervals.

.

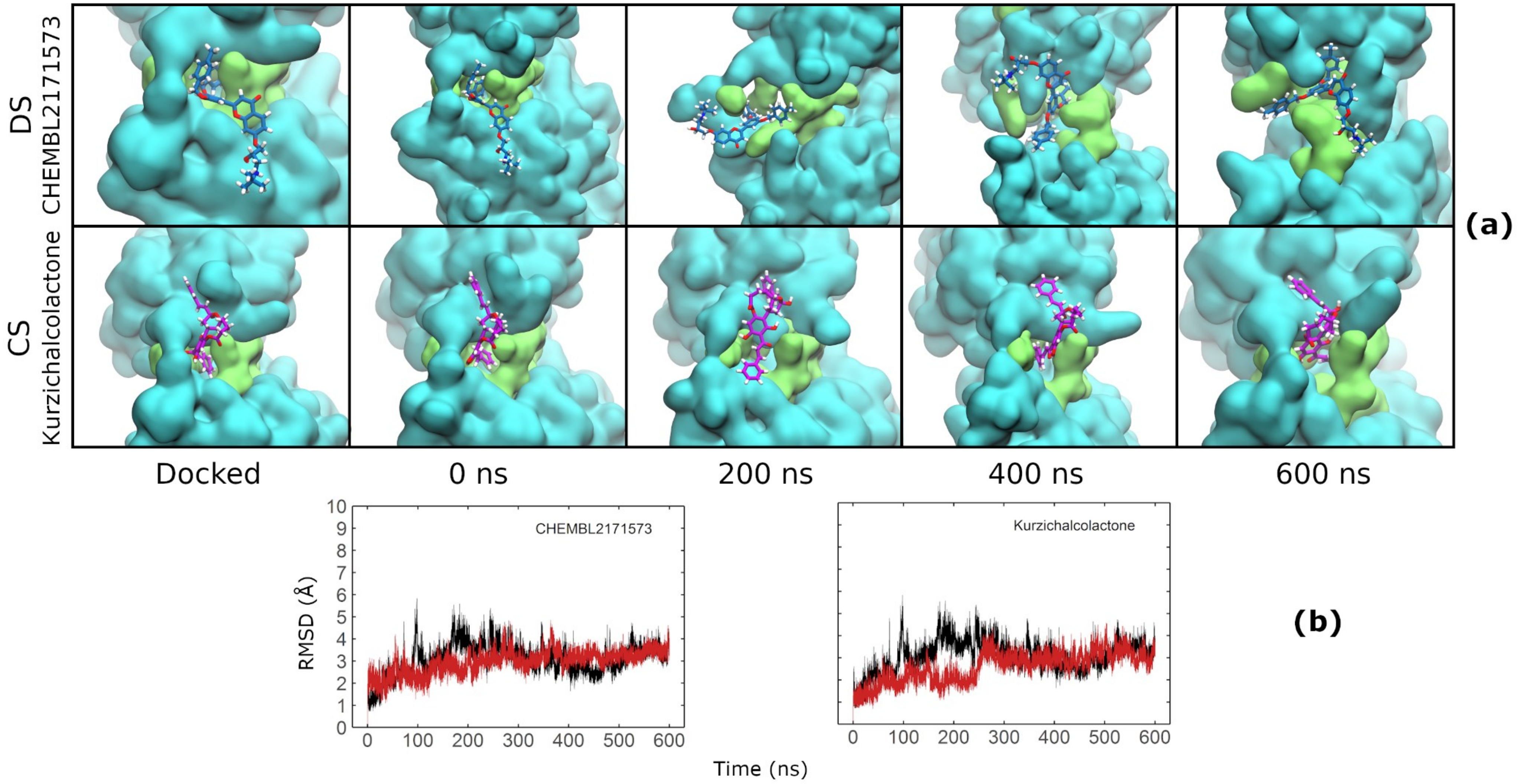

Supplementary Figure 4. Ligand-induced conformational changes in region A by kurzichalcolactone and CHEMBL2171573. (a) Snapshots of molecular dynamics simulations were taken each 200 ns. Residues from Region A are shown in green surface while the rest of the protein is shown in cyan. Ligand backbones are colour-coded according to their initial binding site: blue for dimerization site and magenta for cryptic site. Other atoms follow the CPK colouring convention. **(b)** Ligand-induced RMSD variations in SBS residues are shown in red. RMSD of the *apo*-monomer structure is provided for comparison (black).

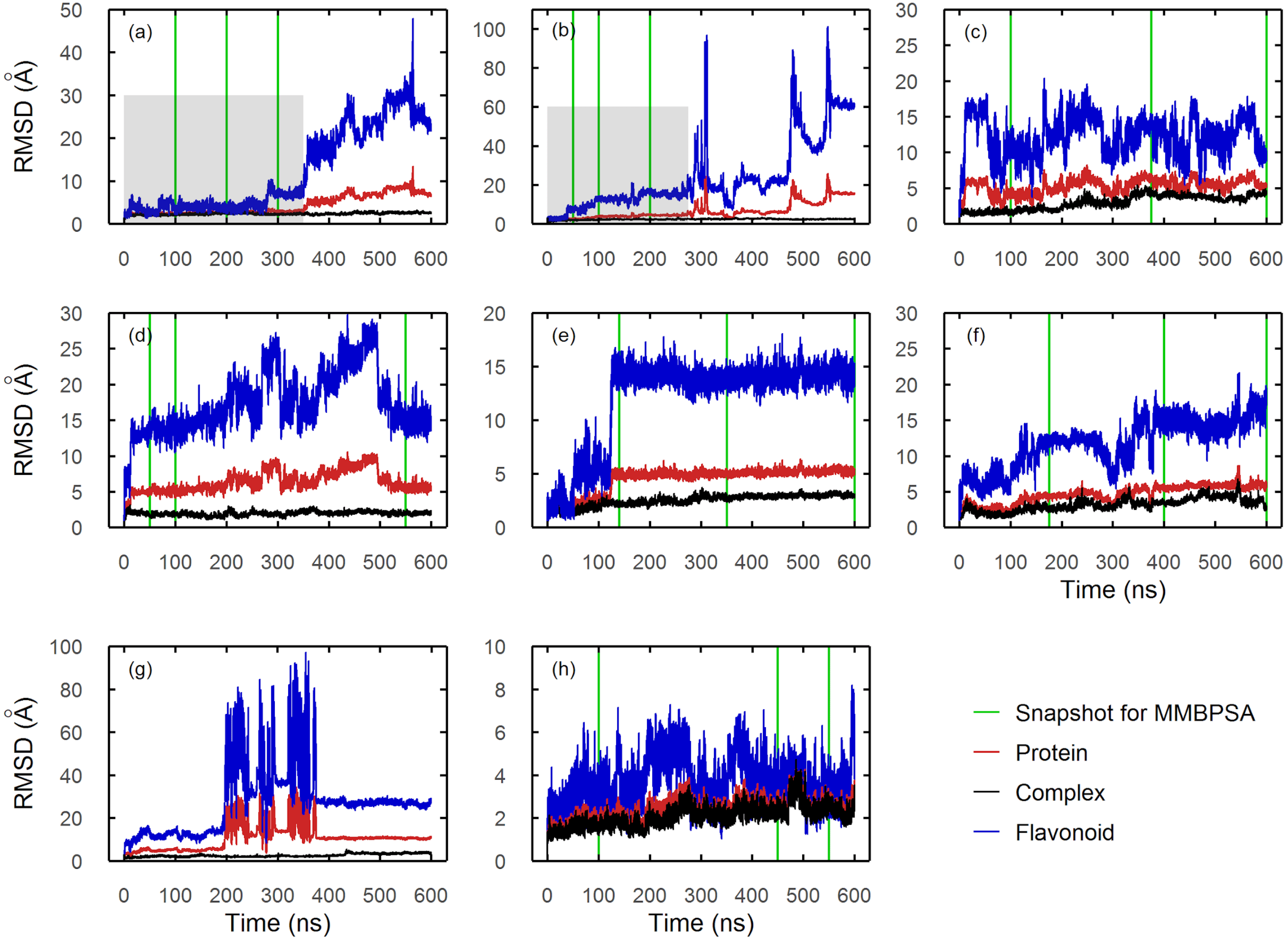

Supplementary Figure 5. RMSD plots of flavonoid-M^{PRO} non-selected ligands along molecular dynamics simulations. Ligands not shown in Figure 3 are depicted here. RMSD of the flavonoid, protein and complex is shown in blue, red, and black, respectively. Green vertical lines reflect snapshots taken for further analysis with MMPBSA. The grey box indicates the time in which the ligand is interacting with the protein. In plots without a grey box, the ligand never detaches from the protein. **SBS: (a)** licorice glycoside E (detaches at 350 ns), **(b)** taxifolin 3'- (6''-phenylacetylglucoside) (detaches at 275 ns). The lowest RMSD and lowest fluctuations within the RT in SBS pertained to licorice glycoside E, which suggest stable binding. **DS: (c)** CHEMBL2171573, **(d)** CHEMBL2171584, **(e)** abyssinoflavanone VI, **(f)** kanzonol E. **CS: (g)** CHEMBL2171578, **(h)** kurzichalcolactone.

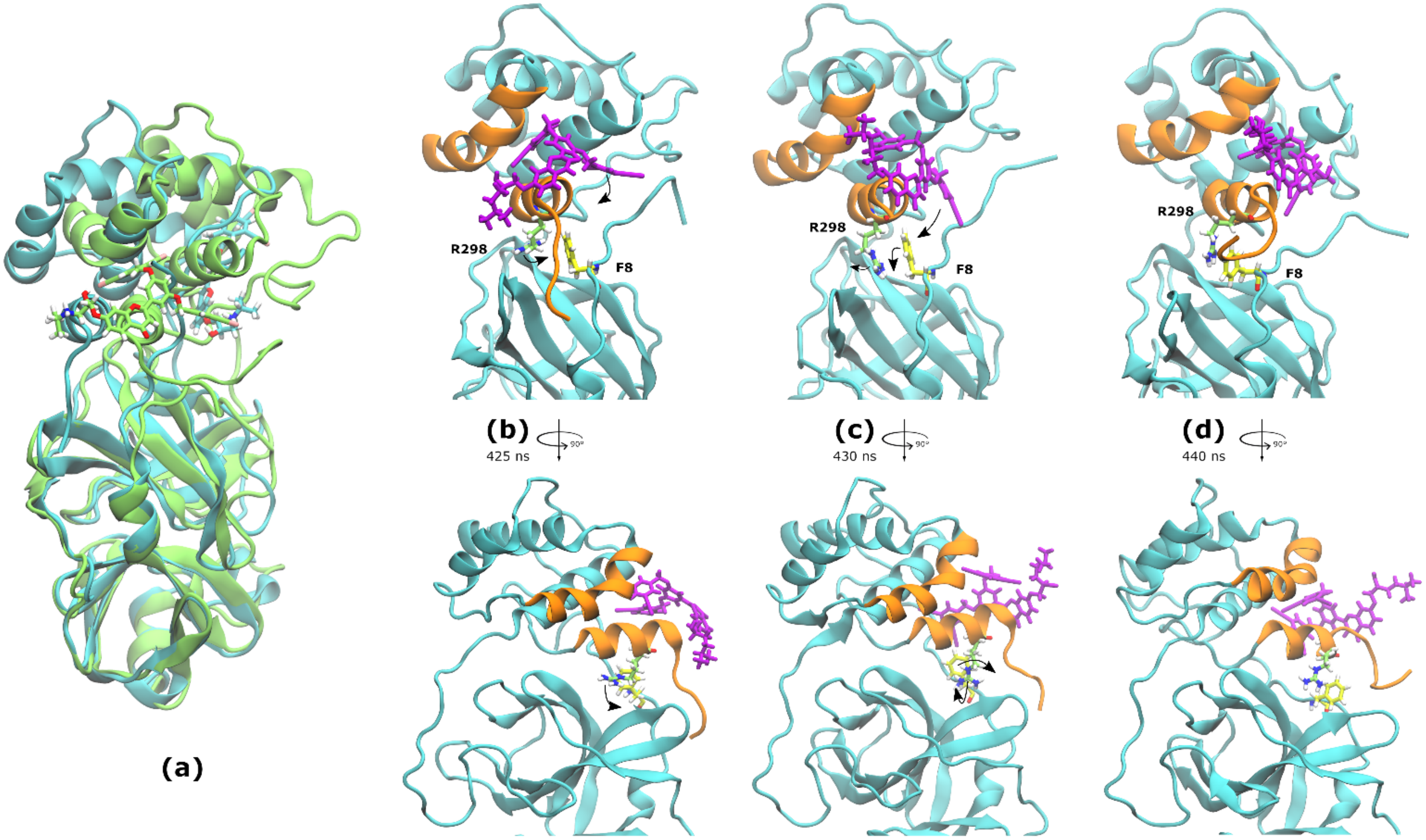

Supplementary Figure 6. Organochloride CHEMBL2171578 bound to region B and promoted the reorientation of M^{PRO} domains I/II, triggering a hinge mechanism. (a) Superimposition of the initial M^{PRO} complex (cyan; frame 0) to the 600 ns snapshot (green; frame 59999) over the domain III. CHEMBL2171578 is shown as sticks. Movements of key residues are shown with black arrows. **(b-d)** MD snapshots detailing domain reorganization and the hinge mechanism. Key residues Phe8 (yellow) and Arg298 (green) are displayed as sticks. No electrostatic interactions were identified. C-terminal helices (residues 244-257 and 293-306) pushed by the ligand's dichlorophenyl rings are coloured orange. One of these rings is sandwiched between said helices. Lower panels are rotated 90° with respect to upper panels to display interactions between domains I/II and III. **(b)** CHEMBL2171578 bound M^{PRO} similar to CHEMBL2171598, engaging in interactions with Phe8 and Arg298. CHEMBL2171578 also induced the rotation of Phe8. **(c)** Phe8's rotation reoriented Phe8 and Arg298 side chains in *anti* conformation. **(d)** After being pushed by an aryl moiety, Phe8 displaced Arg298 and inserted its side chain into the I/II-III cleft. Meanwhile, CHEMBL2171578 adopted its final position in Region B. Of note, CHEMBL2171578 was discarded for potential organochloride-mediated toxicity.

.

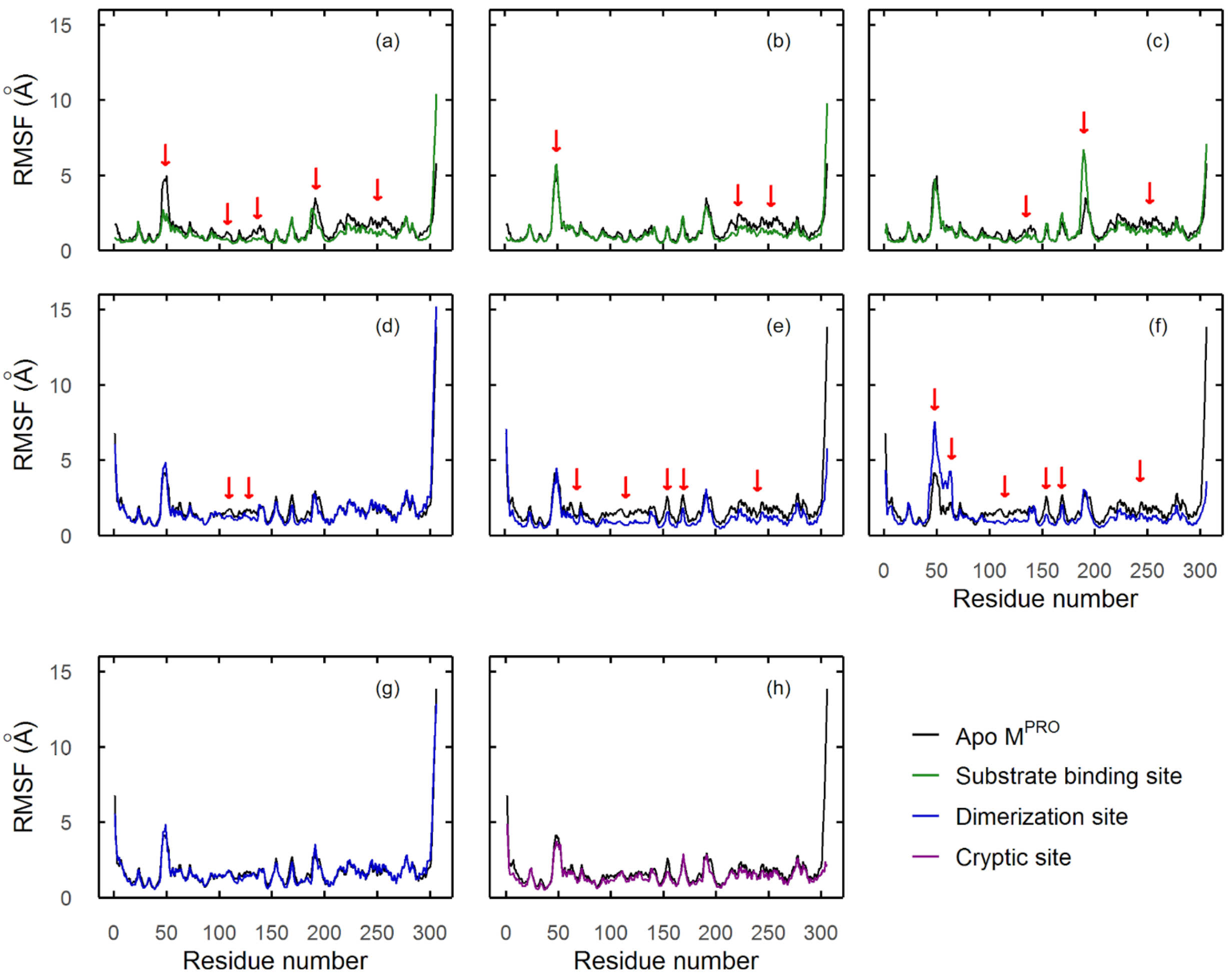

Supplementary Figure 7. RMSF plots of flavonoid-SARS-CoV-2 M^{PRO} non-selected ligands. Ligands not shown in Figure 6 are depicted here. Protein RMSF is shown in green (for SBS), blue (DS) or magenta (CS). *Apo-* dimeric or -monomeric (protomer A) RMSF is shown in black. Red arrows show the most significant differences between *apo-* and *holo-* structures. **SBS: (a)** licorice glycoside E, **(b)** taxifolin 3'- (6"-phenylacetylglucoside), **(c)** X77 (positive control). All SBS ligands affected the protein's flexibility around residue 49. Licorice glycoside E restrained SBS movement, while taxifolin 3'- (6"-phenylacetylglucoside) and X77 increased it. Licorice glycoside E also restrained the movement of residues 105-145. Furthermore, licorice glycoside E and taxifolin 3'- (6"-phenylacetylglucoside) had a long-range effect by reducing the flexibility of domain III residues 200-275. Finally, regarding X77, flexibilization of residues 186-193 plus restrictions on residues 105-145 and 210-275 were observed. **DS: (d)** CHEMBL2171573, **(e)** CHEMBL2171584, **(f)** abyssinoflavanone VI, **(g)** kanzonol E. CHEMBL2171584 and abyssinoflavanone VI restrained the protein movement. An exception was found in residues 45-64 for abyssinoflavanone VI, which showed flexibilization. CHEMBL2171573 showed a small restraint on residues 100-145. **CS: (h)** Kurzichalcolactone.

.

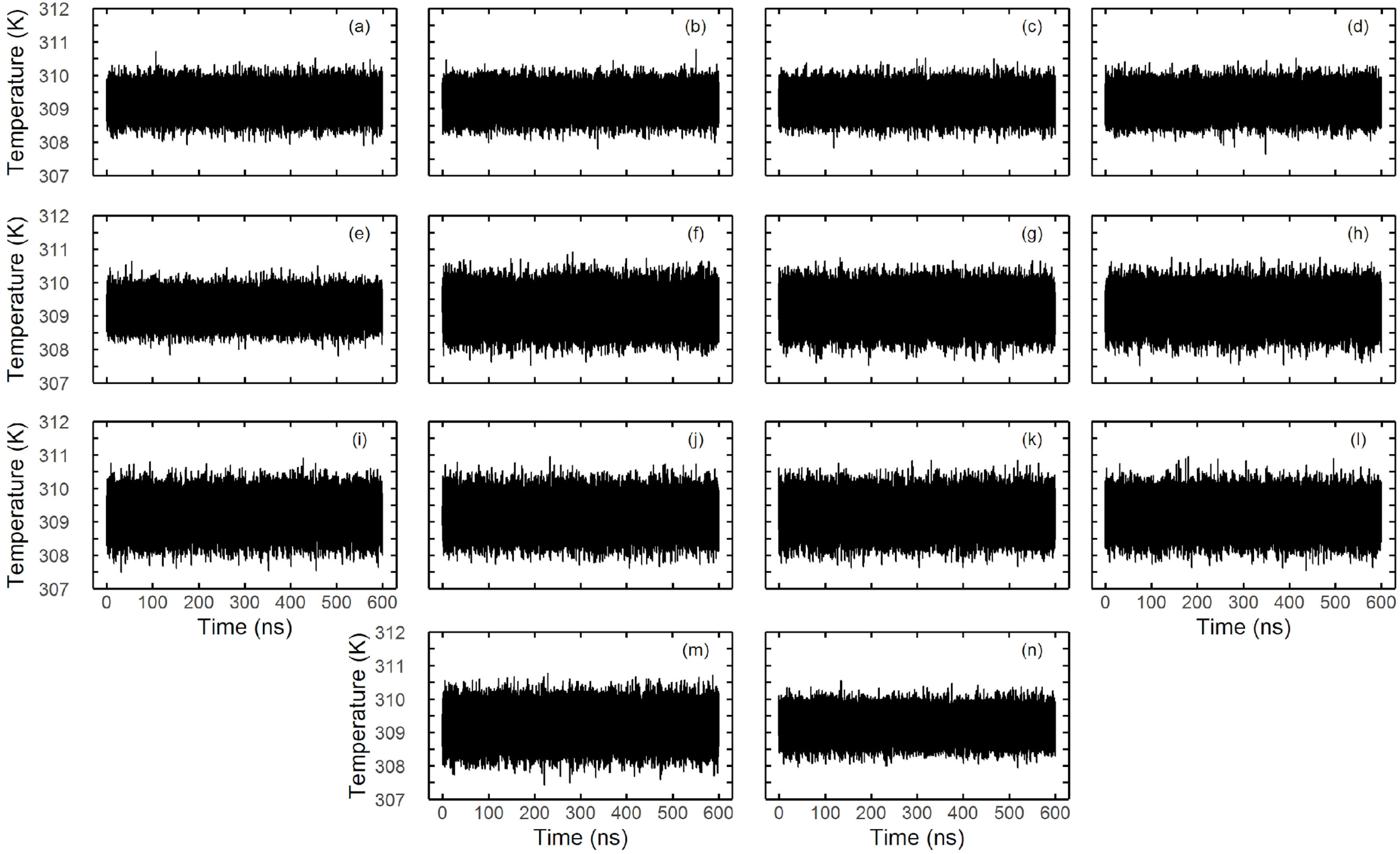

Supplementary Figure 8. Temperature plots of ligands. SBS: (a) licorice glycoside E, **(b)** euchrenone a11, **(c)** dorsilurin E, **(d)** taxifolin 3'- (6"-phenylacetylglucoside), **(e)** X77 (positive control). **DS: (f)** CHEMBL2171573, **(g)** CHEMBL2171584, **(h)** abyssinoflavanone VI, **(i)** sanggenol O, **(j)** kanzonol E. **CS: (k)** CHEMBL2171598, **(l)** kurzichalcolactone. ***Apo*-proteins: (m)** Monomer, **(n)** Dimer.

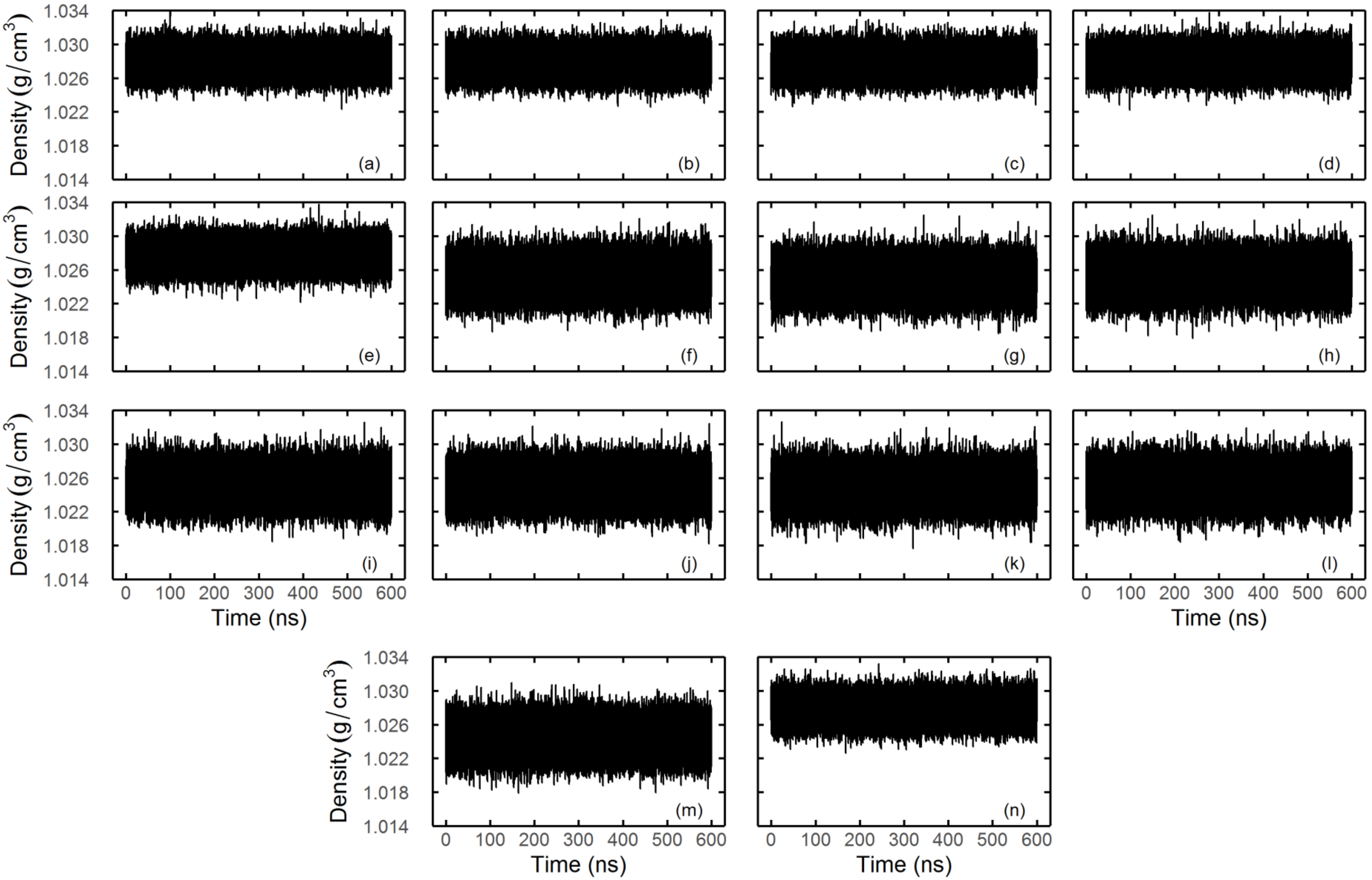

Supplementary Figure 9. Density plots of ligands that remained bound for at least the first 100 ns. SBS: (a) licorice glycoside E, **(b)** euchrenone a11, **(c)** dorsilurin E, **(d)** taxifolin 3'- (6''-phenylacetylglucoside), **(e)** X77 (positive control). **DS: (f)** CHEMBL2171573, **(g)** CHEMBL2171584, **(h)** abyssinoflavanone VI, **(i)** sanggenol O, **(j)** kanzonol E. **CS: (k)** CHEMBL2171598, **(l)** kurzichalcolactone. ***Apo*-proteins: (m)** Monomer, **(n)** Dimer.

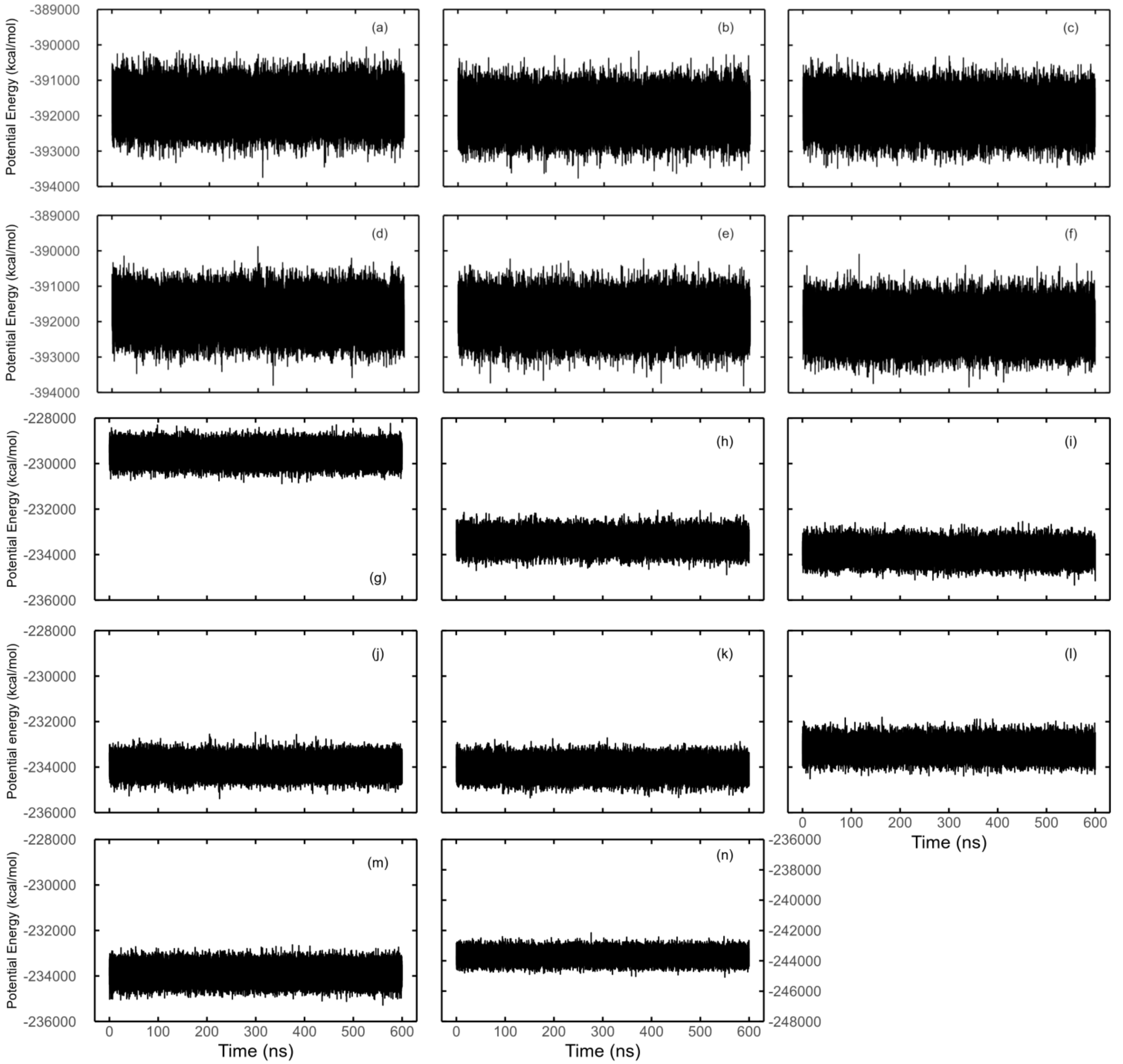

Supplementary Figure 10. Potential energy plots of ligands that remained bound for at least the first 100 ns. SBS: (a) licorice glycoside E, **(b)** euchrenone a11, **(c)** dorsilurin E, **(d)** taxifolin 3'- (6''-phenylacetylglucoside), **(e)** X77 (positive control). **DS: (f)** CHEMBL2171573, **(g)** CHEMBL2171584, **(h)** abyssinoflavanone VI, **(i)** sanggenol O, **(j)** kanzonol E. **CS: (k)** CHEMBL2171598, **(l)** kurzichalcolactone. ***Apo*-proteins: (m)** Monomer, **(n)** Dimer.

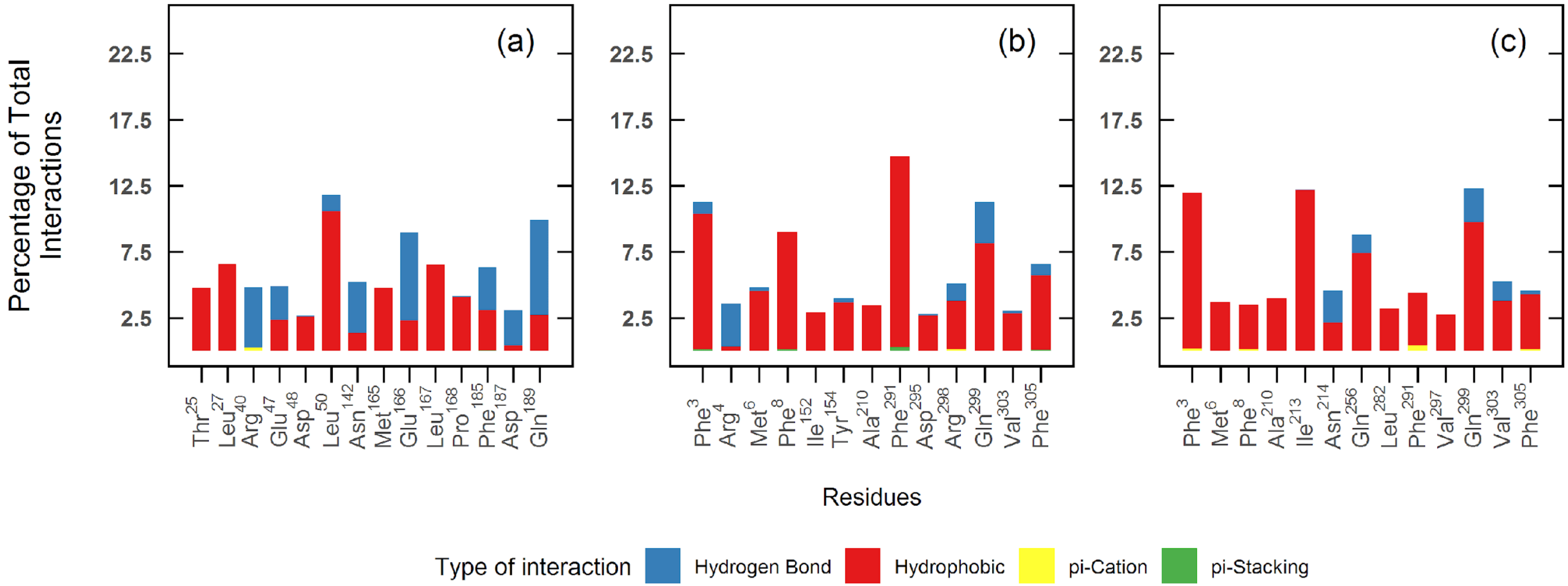

Supplementary Figure 11. Interaction types for non-selected ligands as percentage of total interactions. Ligands not shown in Figure 8 are depicted here. Interaction types are limited to hydrogen bonds (blue), hydrophobic interactions (red), π-Cation contacts (yellow), and π-π stacking contacts (green). Residues with under 2.5% of interactions are not shown. Asterisks (*) indicate residues as belonging to protomer B, the default being protomer A. Note that not all ligands share the same number of interactions, so equal percentages do not mean equal number of interactions. **SBS: (a)** X77 (positive control). **DS: (b)** CHEMBL2171573, **(c)** CHEMBL2171584.

.

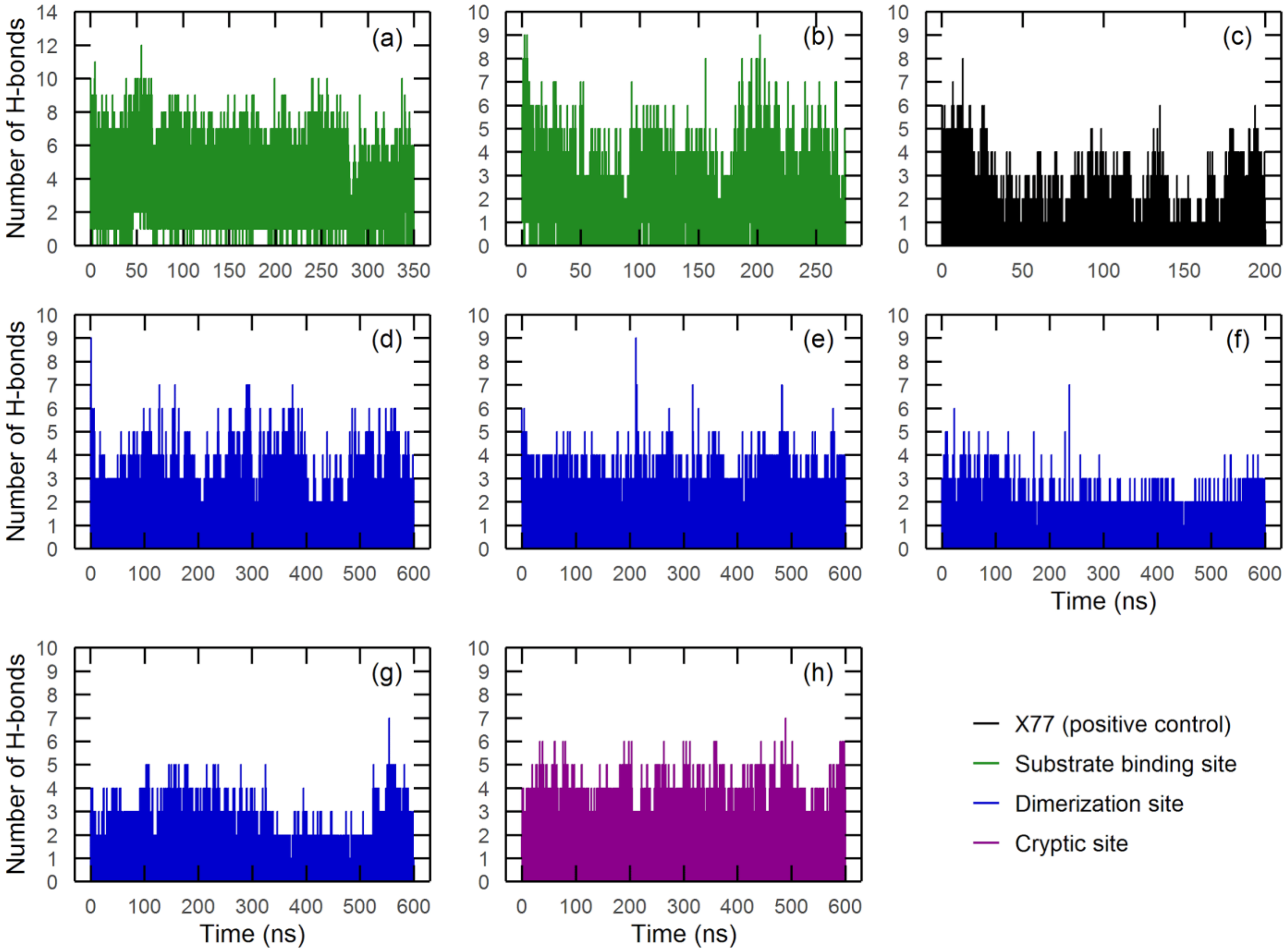

Supplementary Figure 12. Hydrogen bond (H-bond) plot of non-selected ligands. The total number of H-bonds formed between SARS-CoV-2 M^{PRO} and flavonoid ligands is shown by PBS according to previous colour code: green for substrate, blue for dimeric and magenta for cryptic sites. Black represents positive control. **SBS: (a)** licorice glycoside E, **(b)** taxifolin 3'- (6"-phenylacetylglucoside), **(c)** X77 (positive control). Licorice glycoside E held the highest average number of hydrogen bonds at 3.5 (s = 1.59). It is followed by taxifolin 3'- (6"-phenylacetylglucoside) with 1.8 (s = 1.33) and positive control X-77 with 1 (s = 1.07). **DS: (d)** CHEMBL2171573, **(e)** CHEMBL2171584, **(f)** abyssinoflavanone VI, **(g)** kanzonol E. For DS ligands, CHEMBL2171573 holds the second highest average with 1.1 (s = 1.13), followed by CHEMBL2171584 with 0.8 (s = 0.90), kanzonol E with 0.6 (s = 0.81) and abyssinoflavanone VI with 0.6 (s = 0.68). **CS: (h)** kurzichalcolactone. Kurzichalcolactone showed an average of 1.2 H-bonds (s = 1.01) with constant plateaus of 4 H-bonds.

ID	Common name	Site	SMILE	2D-Structure
**FL2F1AGSN001**	Licorice glycoside E	SBS	c(OC(O4)C(OC(O5)C(O)C(COC(=O)c(c76)cnc6cccc7)(C5)O)C(C(C4CO)O)O)(c1)ccc(C(O2)CC(c(c3)c2cc(c3)O)=O)c1	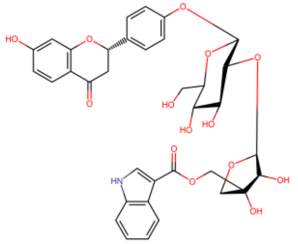
**FL2FALNP0014**	Euchrenone a11	SBS	O=C(c31)CC(c(c5O)cc(c4c5)C=CC(O4)(C)C)Oc1c(CC=C(C)C)c(c2c3O)OC(C=C2)(C)C	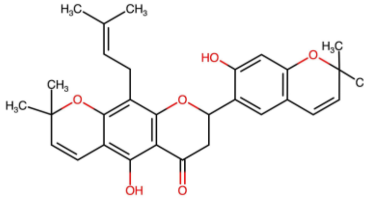
**FL3FQUNP0001**	Dorsilurin E	SBS	c(c54)(O6)c(CCC6(C)C)c(c2c4OC(CC5)(C)C)OC(=c(c3)c(=O)cc(O)c3)C(=C21)CCC(C)(C)O1	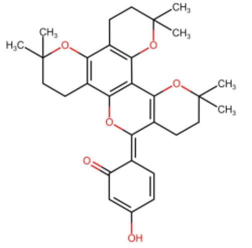
**FL2FA9NC0016**	Kurziflavolactone C	SBS	C(C3=O)CCC(O1)CC(c(c4O)c(cc(O5)c(C(=O)CC(c(c6)cccc6)5)4)O3)CC1(C=Cc(c2)cccc2)O	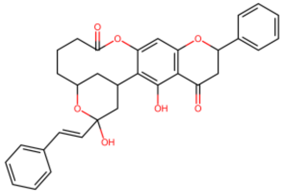
**FL4DACGS0020**	Taxifolin 3'- (6"-phenylacetylglucoside)	SBS	c(c1)(O)cc(c(C(=O)2)c1OC(c(c3)ccc(O)c(OC(O4)C(O)C(C(C(COC(Cc(c5)cccc5)=O)4)O)O)3)C2O)O	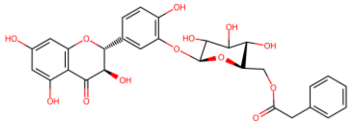

ID	Common name	Site	SMILE	2D-Structure
**FL2FACNP0014**	Abyssinoflavanone VI	DS	c(c5O)c(cc(c45)OC(CC(=O)4)c(c2)c(C1)c(c(O3)c2CCC(C)(C)3)OC(C1O)(C)C)O	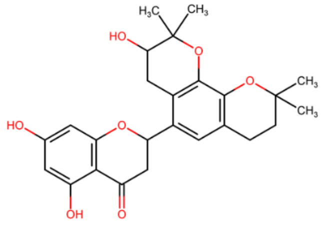
**FL3F1ANP0001**	Kanzonol E	DS	c(c4)(CC=C(C)C)c(cc(c43)OC(=CC3=O)c(c1)cc(C=2)c(OC(C2)(C)C)c1)O	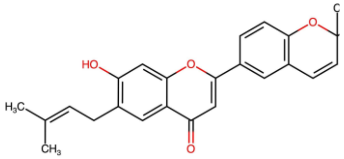
**FL2FALNP0020**	Sanggenol O	DS	c(C(C4)Oc(c5)c(c(cc5O)O)C4=O)(c23)cc(c1c(C=CC(C)(C)O3)2)C=CC(C)(C)O1	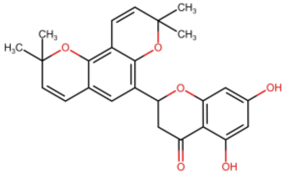
**CHEMBL2171573**	NA	DS	Cc1ccc(COc2cc(OCc3ccc(C)cc3)cc(-c3cc(=O)c4ccc(OCC(O)CNC(C)(C)C)cc4o3)c2)cc1	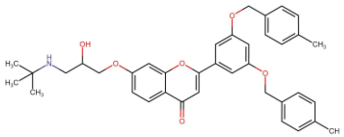
**CHEMBL2171584**	NA	DS	CC(C)(C)NC[C@H](O)COc1ccc2c(=O)cc(-c3cc(OCc4ccccc4)cc(OCc4ccccc4)c3)oc2c1	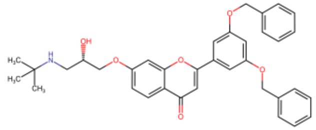

ID	Common name	Site	SMILE	2D-Structure
**CHEMBL2171577**	NA	CS	CC(C)(C)NCC(O)COc1ccc2c(=O)cc(-c3cc(OCc4cc(Cl)cc(Cl)c4)cc(OCc4cc(Cl)cc(Cl)c4)c3)oc2c1	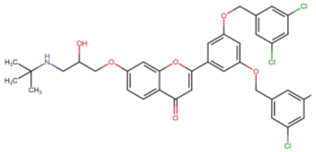
**CHEMBL2171598**	NA	CS	O=c1cc(-c2cc(OCc3ccccc3)cc(OCc3ccccc3)c2)oc2cc(OCC(O)CN3CCN(c4ccccc4)CC3)ccc12	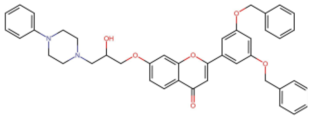
**FL1CA9NC0001**	Kurzichalcolactone	CS	c(c5)ccc(c5)C=CC(C3)(OC(C4)CCCC(Oc(c2C34)cc(c(c(O)2)C(C=Cc(c1)cccc1)=O)O)=O)O	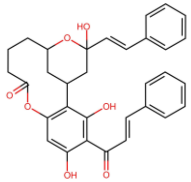
**CHEMBL2171578**	NA	CS	CC(C)NCC(O)COc1ccc2c(=O)cc(-c3cc(OCc4cc(Cl)cc(Cl)c4)cc(OCc4cc(Cl)cc(Cl)c4)c3)oc2c1	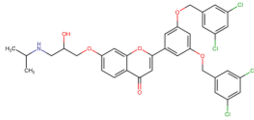
**FL5FAANR0001**	Denticulaflavonol	CS	c(C(O2)=C(C(c(c(O)3)c2cc(c3CC=C(C)CCC(C5(C)4)C(=C)CCC4C(CCC5)(C)C)O)=O)O)(c1)ccc(c1)O	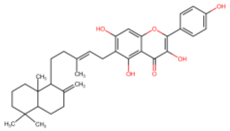

Supplementary Table 1. List and 2D structure of top flavonoid ligands from the exhaustive-ranking screening. The common name, code, SMILE, 2D structure of each flavonoid is indicated, along with the PBS it binds to.

Site	Ligand	Name	Torsions	ΔG	Kd (nM)	Residue	Ligand atom	Distance	Acceptor	Donor	Distance	Type	Residue	Ligand atoms	Distance	Notes
Substrate	**FL3FQUNP0001**	Dorsilurin E	1	-9.51*/ -11.31**	100.87*/ 4.78**	His41	C8	3.31	O35	Gln192	2.06					
						Met49	C7	3.94								
						Met165	C3	3.47								
						Glu166	C21	3.17								
						Leu167	C29	3.99								
						Pro168	C27	3.85								
						Gln189	C3	3.70								
	**FL2FALNP0014**	Euchrenone a11	5	-9.00*/ -10.14**	239.30*/ 34.70**	His41	C24	3.81	Glu166	O35	1.94					
						Met49	C25	3.81	O36	Glu166	2.02					
						Phe140	C30	3.85								
						Leu141	C14	3.41								
						Met165	C15	3.64								
						Asp187	C24	3.27								
	**FL2FA9NC0016**	Kurziflavolactone C	5	-8.88*/ -11.24**	293.25*/ 5.38**	Thr25	C15	3.82	Leu141	O38	1.99	**pi-cation**	His41	C8, C9, C10, C11, C12, C13	5.83	
						Leu141	C23	3.60	O39	Gly143	2.36					
						Asn142	C31	3.79	Gly143	O39	3.50					
						Met165	C3	3.55	O39	Cys145	1.77					
						Glu166	C4	3.76	O34	His163	2.23					
						Val303 (From chain B)	C23, C24	3.34, 3.07								
	**FL2F1AGSN001**	Licorice glycoside E	16	-7.69*/ -9.26**	2201.31*/ 154.06**	Thr25	C15	3.37	O41	Thr26	1.86					
						Met165	C22	3.29	O42	His41	3.08					
						Pro168	C32	3.48	O43	Gly143	2.44					
						Gln189	C23	3.68	O50	Ser144	3.09					
									Ser144	O50	2.74					
									O48	Ser144	2.93					
									O50	His 163	2.04					
									O37	Glu166	3.12					
									O46	Gln192	2.54					
	**FL4DACGS0020**	Taxifolin 3'- (6"-phenylacetylglucoside)	15	-7.21*/-9.27**	4963.68*/ 151.47**	Phe140	C18	3.25	O40	Tyr54	2.58	**pi- stacking**	His41	C22, C23, C24, C25, C26, C27	4.79	pi-Stacking type T
						Met165	C24	3.52	His164	O41	2.49					
						Glu166	C16	3.41	O42	Glu166	1.80					
						Gln189	C23	3.51	Glu166	O42	2.13					
						Gln192	C4	3.77	Asp187	O40	2.18					
									Thr190	O37	1.99					
									O37	Gln192	2.17					

Dimer	**FL2FALNP0020**	Sanggenol O	3	-7.29	4334.61	Phe8	C23	3.41	Met6	O30	2.12					
						Pro9	C6	3.35	O30	Ser113	3.25					
						Thr304	C12	3.70	O30	Gln127	2.60					
						Phe305	C3, C18	3.48, 3.65	O31	Arg298	2.21					
	**CHEMBL2171573**	NA	14	-7.28	4408.66	Met6	C28	3.92	N1	Ser10	3.28	**pi-cation**	Arg298	C25, C26, C27, C28, C29, C31	4.81	Aromatic
						Pro9	C11, C7	3.89, 3.87	O42	Ser10	2.35					
						Val125	C15	3.16	Ser10	O43	1.91					
						Phe291	C30	3.80	O43	Gly11	2.62					
						Asp295	C30	3.53	Glu14	N1	2.61					
						Gln299	C37, C27	3.34, 3.5								
						Phe305	C3	3.62								
	**FL3F1ANP0001**	Kanzonol E	4	-6.92	8112.39	Phe3	C25	3.46	Met6	O29	2.02					
						Met6	C24	3.60	O29	Gln127	3.72					
						Phe8	C4	3.57								
						Pro9	C16	3.29								
						Phe291	C24, C25	3.10, 3.14								
						Asp295	C22	3.07								
						Arg298	C6	3.98								
						Gln299	C25	3.63								
						Thr304	C12	3.45								
						Phe305	C10, C15	3.92, 3.13								
	**CHEMBL2171584**	NA	14	-6.89	8535.30	Pro9	C10, C11	3.67, 3.87	Ser10	N1	1.76	**pi-cation**	Arg298	C18, C19, C20, C21, C22, C23	4.74	Aromatic
						Glu14	C35	3.92	O43	Ser10	2.79					
						Leu115	C37	3.27	Ser10	O43	2.47					
						Pro122	C35	3.42	O42	Ser10	2.58					
						Val125	C3	3.97								
						Arg298	C20	3.87								
						Gln299	C28, C20	3.46, 3.39								
						Phe305	C14	3.49								
	**FL2FACNP0014**	Abyssinoflavanone VI	4	-6.83	9448.42	Pro9	C7	3.66	Met6	O30	2.04	**pi-stacking**	Phe8	C18, C19, C20, C21, C22, C23	4.18	pi-Stacking type P
						Thr304	C8	3.76	O30	Ser113	3.23					
						Phe305	C25	3.68	O30	Gln127	2.63					
									O31	Arg298	2.18					

						Lys5	C41	3.48	Lys5	O50	2.01					
						Met6	C21	3.72	O49	Ala7	2.97					
						Pro9	C4	3.14	Val125	N1	2.11					
	**CHEMBL2171598**	NA	14	-10.59	16.19	Tyr126	C44	3.88								
						Asp295	C22	3.54								
						Gln299	C21, C30	3.63, 3.26								
						Phe305	C15	3.55								
						Tyr237	C32, C37	3.93, 3.96	O41	Lys5	1.73	**Halogen**	Asp197	Donor: Cl46	3.29	Aceptor: O1911
						Asn238	C35	3.7	O38	Thr199	2.26					
						Tyr239	C33	3.78	O42	Leu287	2.18					
	**CHEMBL2171577**	NA	14	-10.51	18.54	Leu286	C7, C21, C23	3.47, 3.21, 3.14	Glu288	N1	2.92					
						Leu287	C7	3.95	Glu288	O41	2.02					
									O40	Asp289	2.44					
						Lys137	C7	3.69	O40	Lys5	1.97	**pi-cation**	Arg131	C4, C5, C6, C7, C8, C9	4.03	Aromatic
						Thr199	C15	3.85	O41	Gln127	2.28					
						Tyr237	C27	3.65	O39	Lys137	1.97					
						Tyr239	C28	3.21	Asp197	O39	2.13					
**Cryptic**	**FL5FAANR0001**	Denticulaflavonol	10	-10.44	20.87	Leu272	C27	3.28	Glu288	O40	1.94					
						Leu286	C21	3.63	O37	Glu288	2.69					
						Leu287	C22, C18	3.68, 3.52	O37	Asp289	1.82					
									O38	Asp289	2.05					
									O40	Glu290	3.47					
						Phe8	C29	3.82	Gly2	O36	2.04	**pi-cation**	Arg298	C29, C31, C28, C30, C27, C32	4.55	Aromatic
	**FL1CA9NC0001**	Kurzichalcolactone	8	-10.23	29.79	Ile213	C22	2.95	Gln299	O37	2.03					
						Asp295	C31	3.75	Val303	O39	1.86					
						Arg298	C26	3.40	O38	Val303	2.25					
						Gln299	C2	3.82								
						Thr199	C9	3.56	O37	Arg131	2.80	**Halogen**	Asp197	Donor: Cl44	3.75	Acceptor: O1910
						Tyr237	C25	3.62	O37	Thr199	2.08					
						Asn238	C27	3.44	O42	Leu287	2.15					
	**CHEMBL2171578**	NA	14	-9.97	46.28	Tyr239	C7	3.66	Glu288	N1	3.14					
						Leu286	C22, C16, C3	3.54, 3.70, 3.67	Glu288	O40	1.91					
						Leu287	C3, C5	3.61, 3.57								

* NSBS
** ISBS

Supplementary Table 2. Comprehensive list of protein-ligand interactions. Top 5 binding energy flavonoids are listed for each PBS. Interactions are according to PLIP of ligand binding against (ISBS). ΔG and values for the substrate binding site is marked with * for the normal docking (NSBS) procedure and ** for the induced-fit docking (ISBS). Note that both ligand and protein can participate as donor or acceptor in hydrogen bonds. Thus, ligands are represented with their atom name and number, and protein residues are portrayed as their name and number. For the SBS, unless otherwise stated, interacting residues of the M^{PRO} are from protomer A.

Ligand	Fast-Ranking Screening		Exhaustive-Ranking Screening	
	SBS			
	NSBS	ISBS	NSBS	ISBS
FL2F1AGSN001	23	29	22	29
FL2FALNP0014	44	58	6	14
FL3FQUNP0001	44	29	2	2
FL2FA9NC0016	68	75	8	3
FL4DACGS0020	23	58	26	28
	DS			
FL2FACNP0014	24		5	
FL3F1ANP0001	12		3	
FL2FALNP0020	1		1	
CHEMBL2171573	24		2	
CHEMBL2171584	43		4	
	CS			
CHEMBL2171577	27		2	
CHEMBL2171598	27		1	
FL1CA9NC0001	27		4	
CHEMBL2171578	54		5	
FL5FAANR0001	54		3	

Supplementary Table 3. Comparison of ligand ranking between sites and stages. Selected ligands and their corresponding positions in both the fast- and exhaustive-ranking stages. Ligands which consistently landed on the top 40 for the fast-ranking stage are highlighted.

ID	Common Name	PAIN structure	Source proposing the molecule for SARS-CoV-2 M^{PRO} inhibition	Source concluding or suggesting the molecule as PAINs
CHEMBL31574	fisetin	whole molecule	24	52, 54
FL63AGNS0004	gallocatechin 3-O-gallate	(+)-catechin	23	27
FL5FAGGS0004	myricitrin	myricetin	20	27
FL5FACNS0001	quercetin	whole molecule	51	27, 52, 53, 54
FL5FACGS0006	quercetin 3-rhamnoside	quercetin	22	27
FL5FACGL0001	quercetin 3-O-β-D-glucoside	quercetin	20	27
FL5FAGGL0003	myricetin 3-rutinoside	myricetin	22	27
FL5FACGL0013	rutin	whole molecule	20, 21, 26	27

Supplementary Table 4. PAIN flavonoids present in recent literature. IDs and common names of the flavonoids previously reported as SARS-CoV-2 M^{PRO} inhibitors but now reported as PAINs. The source studies for their proposals as such are provided, as well as the probable mechanisms and moieties responsible for their interference with assays.

	Fast-Ranking Screening (AutoDock Vina)		Exhaustive-Ranking Screening (AutoDock GPU)	
**Site**	**Dimensions (Å)**	**Coordinates**	**Dimensions (Å)**	**Coordinates**
NSBS	35 × 35 × 35	12.339, 1.287, 23.152	35.25 × 35.25 × 35.25	12.339, 1.287, 23.152
ISBS	36 × 35 × 35	-15.117, 14.564, 67.870	35.25 × 35.25 × 35.25	-15.117, 14.564, 67.870
DS	22 × 28 × 36	1.738, -3.380, 4.457	22.5 × 30 × 37.5	1.738, -3.380, 4.457
CS	30 × 40 × 30	9.104, 12.126, -6.685	30.375 × 40.5 × 30.375	9.104, 12.126, -6.685

Supplementary Table 5. Site dimensions and coordinates for each box. Site dimensions and coordinates are shown. For the case of SBS boxes, the distinction is shown between ISBS and NSBS.

Supplementary Dataset 1. Compounds identified as PAINs using FAFDrugs4 and removed from the original 6697 database. Includes flavonoid common name, code (per database) and SMILEs. Nonetheless, there are two general problems which need to be addressed in future flavonoid based VSs. First, virtual filtering tools are liable to limitations such as the size or actuality of their parent libraries[2]. Second, the existence of small molecule FDA-approved drugs which trigger these filters should caution against premature exclusion of alleged PAINs[3].

Supplementary Dataset 2. **Final, PAIN-filtered 4858-compound database.** Includes flavonoid code (per database), common name, molecular weight (g/mol), molecular formula, SMILEs and number of torsions.

Supplementary Dataset 3. Fast-ranking screening results per PBS. (a) NSBS (PDB ID: 6YB7)[4], mean = -7.39 kcal/mol, top 100 mean = -8.89 kcal/mol; **(b)** ISBS (PDB ID: 6LU7)[1], mean = -8.15 kcal/mol, top 100 mean = -10.05 kcal/mol. **(c)** DS, mean = -6.53 kcal/mol, top 100 mean = -8.07 kcal/mol. **(d)** CS, mean = -7.42 kcal/mol, top 100 mean = -9.12 kcal/mol. List includes flavonoid code, binding mode, binding energy (kcal/mol), RMSD lower bound, RMSD upper bound, and flavonoid common names.

Supplementary Dataset 4. Exhaustive-ranking screening results per PBS. (a) NSBS (PDB ID: 6YB7)[4], mean = -5.58 kcal/mol, top 5 mean = -9.52 kcal/mol; **(b)** ISBS (PDB ID: 6LU7)[1], mean = -7.98 kcal/mol, top 5 mean = -11.51 kcal/mol. **(c)** DS, mean = -4.37 kcal/mol, top 5 mean = -7.04 kcal/mol. **(d)** CS, mean = -6.56 kcal/mol, top 5 mean = -10.35 kcal/mol. List includes ligand cluster, lowest binding energy (kcal/mol), run number, mean-binding energies (kcal/mol), number of binding modes (poses) in the cluster, normalized number of binding modes (poses) in the cluster, normalized binding energies (kcal/mol), 2Dscore, flavonoid code and flavonoid common names.

Supplementary Methods. Last discarded ligands.

After reviewing the molecular dynamics of the ligands which made it to that stage, a last filtering step was taken.

Regarding SBS, licorice glycoside E and taxifolin 3’- (6”-phenylacetylglucoside) had positive binding energies (Figure 7aiv and av, respectively), so they were discarded. Regarding DS excluded ligands, abyssinoflavanone VI had the highest binding energy (Figure 7biv) and fewest hydrogen bonds (Supplementary Figure 12f). Additionally, kanzonol E did not impact RMSF (Supplementary Figure 7g) and had relatively few hydrogen bonds (Supplementary Figure 12g). Lastly, for CS ligands, kurzichalcolactone was excluded because it was unstably bound to region A without a drastic impact on SBS (Supplementary Figure 4b). Besides, its binding energy (Figure 7cii) was significantly higher than the one of CHEMBL2171598.

Thus, selected ligands for interaction analysis were dorsilurin E and euchrenone a11 for SBS; sanggenol O, CHEMBL2171573 and CHEMBL2171584 for DS and CHEMBL2171598 for CS. Positive control was also included for comparison. Then, these compounds passed through an in-house pipepline that identified and classified their interactions formed with M^{PRO} along MD.

Supplementary Materials. References.

1. Jin, Z. et al. Structure of Mpro from SARS-CoV-2 and discovery of its inhibitors. Nature 582, 289–293 (2020).

2. Capuzzi, S. J., Muratov, E. N. & Tropsha, A. Phantom PAINS: problems with the utility of alerts for p an-a ssay in terference compound S. J. Chem. Inf. Model. 57, 417–427 (2017).

3. Baell, J. B. & Nissink, J. W. M. Seven year itch: Pan-assay interference compounds (PAINS) in 2017—utility and limitations. ACS Chem. Biol. 13, 36–44 (2018).

4. Owen, C. D. et al. 6YB7: SARS-CoV-2 main protease with unliganded active site (2019-nCoV, coronavirus disease 2019, COVID-19). Protein Data Bank https://www.rcsb.org/structure/6yb7 (2020).
